# Exploratory analysis of biological age measures in a remyelination clinical trial

**DOI:** 10.1093/braincomms/fcaf032

**Published:** 2025-01-29

**Authors:** Christopher E McMurran, Ermelinda de Meo, Nick G Cunniffe, J William L Brown, Ferran Prados, Baris Kanber, James H Cole, Alasdair J Coles, Sara Hägg, Declan T Chard

**Affiliations:** Department of Clinical Neurosciences, University of Cambridge, Cambridge, CB2 0AH, UK; Department of Neuroinflammation, NMR Research Unit, Queen Square Multiple Sclerosis Centre, University College London (UCL) Queen Square Institute of Neurology, London, WC1B 5EH, UK; Department of Medical Epidemiology and Biostatistics, Karolinska Institutet, Stockholm, SE-171 77, Sweden; Department of Neuroinflammation, NMR Research Unit, Queen Square Multiple Sclerosis Centre, University College London (UCL) Queen Square Institute of Neurology, London, WC1B 5EH, UK; Department of Clinical Neurosciences, University of Cambridge, Cambridge, CB2 0AH, UK; Department of Clinical Neurosciences, University of Cambridge, Cambridge, CB2 0AH, UK; Department of Neuroinflammation, NMR Research Unit, Queen Square Multiple Sclerosis Centre, University College London (UCL) Queen Square Institute of Neurology, London, WC1B 5EH, UK; Department of Neuroinflammation, NMR Research Unit, Queen Square Multiple Sclerosis Centre, University College London (UCL) Queen Square Institute of Neurology, London, WC1B 5EH, UK; Department of Medical Physics and Biomedical Engineering, Centre for Medical Image Computing, University College London, London, WC1V 6LJ, UK; eHealth Center, Universitat Oberta de Catalunya, Barcelona 08018, Spain; Department of Neuroinflammation, NMR Research Unit, Queen Square Multiple Sclerosis Centre, University College London (UCL) Queen Square Institute of Neurology, London, WC1B 5EH, UK; Department of Computer Science, Centre for Medical Image Computing, University College London, London, WC1V 6LJ, UK; Dementia Research Centre, Queen Square Institute of Neurology, University College London, London, WC1N 3AR, UK; Department of Clinical Neurosciences, University of Cambridge, Cambridge, CB2 0AH, UK; Department of Medical Epidemiology and Biostatistics, Karolinska Institutet, Stockholm, SE-171 77, Sweden; Department of Neuroinflammation, NMR Research Unit, Queen Square Multiple Sclerosis Centre, University College London (UCL) Queen Square Institute of Neurology, London, WC1B 5EH, UK; National Institute for Health Research (NIHR) University College London Hospitals (UCLH) Biomedical Research Centre, London, W1T 7DN, UK

**Keywords:** remyelination, clinical trial, biological age, brain age, MRI

## Abstract

Enhancing CNS myelin repair (remyelination) is a promising strategy to prevent neurodegeneration and associated progressive disability in multiple sclerosis. Remyelination becomes inefficient with older chronological age, but the relationship between measures of biological age and remyelination has not been previously described in a clinical cohort. Here, we investigated two measures of biological age amongst participants of the Cambridge Centre for Myelin Repair One trial of bexarotene: MRI brain age (BA_MRI_) and a blood-based biological age (BA_Blood_). In people with radiologically stable multiple sclerosis (*n* = 44 of 49 total participants), we found that treatment with bexarotene, along with promoting remyelination, was associated with significant decrease in MRI brain age [−1.98 years, 95% confidence interval (CI) [−3.75, −0.21 years] versus placebo over 6 months, *P* = 0.034]. Whilst BA_MRI_ increased as expected during the trial in the placebo group (+0.92 years, CI [−0.41, 2.26]), the brain MRIs of participants treated with bexarotene appeared on average 11 months younger at the end compared to the start of the trial (−0.93 years, CI [−2.02, 0.17]). The effect of bexarotene on BA_MRI_ was associated with its remyelinating activity in cortical grey matter lesions (*β* = 0.25% units (pu)/year, CI [0.03, 0.46], *P* = 0.023) and brainstem lesions (*β* = 0.24 pu/year, CI [0.09, 0.39], *P* = 0.003). We also observed some regional trends that the remyelinating response to bexarotene was linked with measures of biological age at baseline. For example, after adjustment for chronological age, remyelination of brainstem lesions assessed by magnetization transfer ratio was reduced by 0.06 pu for each year increase in BA_MRI_ (CI [0.00, 0.13], *P* = 0.058) and 0.02 pu for each year increase in BA_Blood_ (CI [−0.01, 0.05], *P* = 0.17). This is, to the best of our knowledge, the first demonstration that MRI brain age can be therapeutically modulated by a drug in people with a neurological disorder. Overall, these findings highlight that beyond chronological age, biological age may also influence the potential for repair and should be considered when developing treatments for multiple sclerosis.

## Teaser

McMurran *et al*. investigated how brain repair relates to biological age in multiple sclerosis. People receiving a remyelination-promoting drug had a relative reduction in MRI brain age, alongside evidence that baseline biological age influences remyelination in some regions. This emphasizes the importance of thinking beyond chronological age in multiple sclerosis.

## Graphical Abstract

**graphical abstract:**
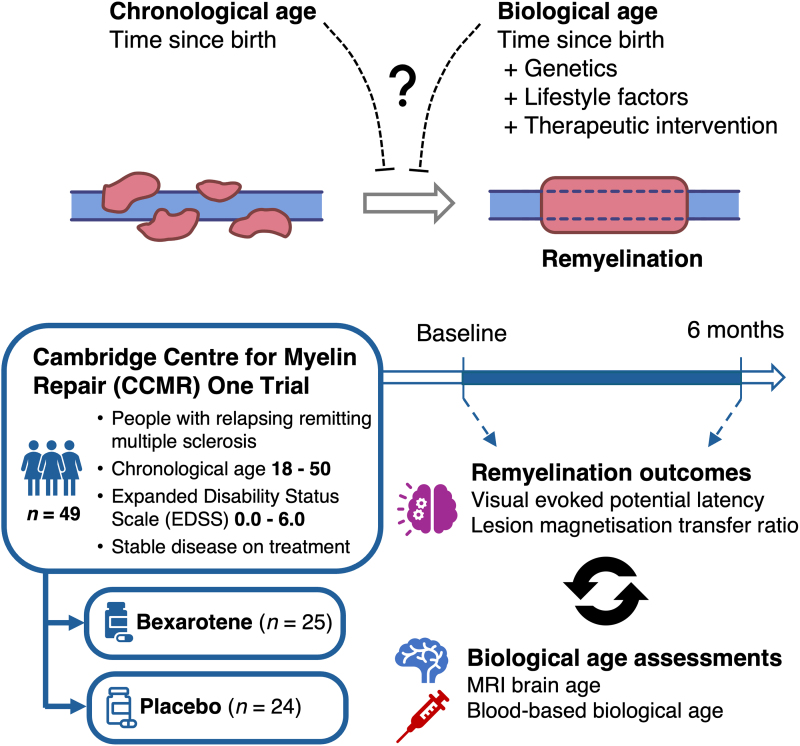

## Introduction

Age is one of the most important predictors of disability accumulation in multiple sclerosis. Although multiple sclerosis typically manifests in young adulthood, advancing age is associated with conversion from relapsing-remitting to secondary progressive multiple sclerosis,^1^ diminished post-relapse recovery^2^ and lower disease-modifying therapy efficacy.^3^ An important underlying mechanism is thought to be the failure of endogenous remyelination with advancing age,^4,5^ and enhancing this process is a priority for the development of future therapies in multiple sclerosis.^6^

In a cohort of people with relapsing-remitting multiple sclerosis (RRMS), we have previously demonstrated that the therapeutic response to bexarotene, a remyelination-promoting drug, diminished with increasing chronological age (CA, calendar time since birth).^7^ Specifically, the effects of bexarotene on visual-evoked potential (VEP) latency reduction and deep grey matter (GM) magnetization transfer ratio (MTR) measures were only significant amongst participants up to the age of 42–43.

The ageing process can be measured readily as CA, but heterogeneity in ageing between individuals can be better described by biological age (BA). Biological ageing can be assessed using a range of different biomarkers, including brain MRI data^8^ or routine clinical blood results.^9^ These approaches allow us to consider how people age with different trajectories and the factors underlying this variability.^10^ For example, people with multiple sclerosis have an MRI brain age a decade higher on average than healthy age-matched controls,^11^ with higher brain age predicting greater disability in the future.^12^ Similarly, a BA measure based on routine clinical biomarkers is higher than CA for people with MS and correlated with disability outcome measures in a prospective cohort study.^9^ However, despite much remyelination research in preclinical settings focused on the modifiable effects of age,^13-15^ the relationship between remyelination and BA measures in people has not been previously addressed.

The Cambridge Centre for Myelin Repair (CCMR) One trial was a two-centre, randomized, double-blind, placebo-controlled, phase 2a trial of bexarotene for people with RRMS.^16^ Bexarotene at this dose was associated with unacceptable toxicity in the study population, but nevertheless, the data generated provide a window into the biology of remyelination in humans. In the present analysis, we derive two measures of BA for participants of the CCMR One cohort: an MRI-based brain age^8^ (BA_MRI_) and a blood BA based on routine clinical biomarkers (BA_Blood_)^17,18^ and use these measures to assess the relationship between BA and remyelination in people with multiple sclerosis.

## Materials and methods

### Cambridge Centre for Myelin Repair One trial

The CCMR One trial was carried out in accordance with the Declaration of Helsinki and was approved by London Westminster National Research Ethics Service Committee (15/LO/0108). The full protocol and results are published elsewhere.^16^ In brief, patients aged 18–50 with RRMS on a stable dose of dimethyl fumarate, with Expanded Disability Status Scale (EDSS) 0.0–6.0, were recruited at two centres: Cambridge and Edinburgh. Participants were randomized to receive either oral bexarotene (300 mg/m^2^ up to 750 mg per day; *n* = 26) or equivalent placebo tablets (*n* = 26). All participants were assessed with MRI brain and full-field VEP testing at baseline and at 6 months. Data were analysed by intention to treat for all those completing both baseline and 6-month assessments (*n* = 49 people; 28 (57%) female). For VEP analyses, we focused on eyes with prolonged baseline P100 latency (>118 ms; *n* = 51 eyes from 28 people). This cut-off represents a greater degree of demyelination at baseline and was based on previous work that showed the most robust effects of remyelinating therapies amongst these eyes.^7,16,19^ To reduce confounding effects related to simultaneous inflammation and/or demyelination, we also carried out sensitivity analysis excluding eyes with a clinical history of optic neuritis in the preceding 5 years (leaving *n* = 42 eyes from 26 people). For the same reason, we analysed changes in BA_MRI_ amongst only those without new MRI lesions during the trial (*n* = 44 people) as well as in the full population. Sample sizes for each analysis are summarized in Supplementary Table 1. The CA range of patients receiving bexarotene was 29–49 (mean 40.4) and the placebo group 25–49 (mean 38.0).

#### MRI acquisition

Both study sites used a Siemens 3T Prisma^fit^ scanner (Siemens, Erlangen, Germany) with 20-channel head–neck coils. Scans included the following sequences: 3DT1 (for volumetric measures and segmentation: 1 × 1 × 1 mm, TR = 2400 ms, TE = 2.99 ms, flip angle = 8°), interleaved proton-density/T2-weighted scans (to identify and contour white matter (WM), deep GM (DGM) and cortical GM (CGM) T2 hyperintense lesions: 1 × 1 × 3 mm, TR = 3050 ms, TE = 31/82 ms), fluid-attenuated-inversion recovery (FLAIR, to aid lesion identification: 1 × 1 × 3 mm, TR = 9500 ms, TE = 123 ms) and 3D magnetization transfer imaging (to calculate MTR maps: 1 × 1 × 1 mm, TR = 35 ms, TE = 4.07/9.49 ms, flip angle 9°). Three different observers blinded to clinical and treatment details performed lesion identification, contouring and checking. Consistent with the original trial, baseline lesion masks were overlaid onto the 6-month scans so that the same tissue was examined at both time points (although this did not account for dynamic effects from shrinking or expanding lesions). T1 volumetric scans were filled using a lesion-filling patch-based method^20^ and then segmented into regions using the geodesic information flows,^21^ to automatically classify lesions by location. Further details are available in the original trial manuscript.^16^ The MTR maps at baseline and 6-month follow-up were calculated directly as follows:


(1) $$\mathrm{MTR}=((MT_{\mathrm{off}}-\;MT_{\mathrm{on}})/MT_{\mathrm{off}})\;\times \;100$$

We focused our analysis on lesions in CGM, DGM and the brainstem due to previous work showing that bexarotene significantly enhanced remyelination in these three regions,^16^ with the effect in DGM being significantly attenuated by increasing CA.^7^

### Biological age measures

#### MRI brain age

BA_MRI_ calculation followed our previously established brainageR protocol available at www.github.com/james-cole/brainageR.^22,23^ Briefly, T1-weighted images were segmented into GM, WM and cerebrospinal fluid and normalized using the SPM12 software package (www.fil.ion.ucl.ac.uk/spm/software/spm12/). Visual quality control was conducted to verify segmentation accuracy, and all images were included. The normalized images were loaded into R using the *RNfiti* package, vectorized and GM, WM and CSF probability values were masked (using 0.3 in the average image from the brainageR-specific template, derived from *n* = 200 scans) and then concatenated. The brainageR model was trained on *n* = 3377 healthy individuals (mean age 40.6 years, SD 21.4) from seven publicly available data sets. A principal component analysis (PCA) was run and the top 80% of variance retained. The rotation matrix of the training set PCA was applied to the CCMR One data, and the output was used to predict the CA of each scan, using Gaussian process regression with radial basis function kernel and default hyperparameters. This approach to calculating MRI brain age has been tested and validated in several large independent data sets (www.github.com/james-cole/brainageR).^24^

#### Blood-based biological age

BA_Blood_ was generated using the Klemera–Doubal method^17^ within the *BioAge* package in *R*.^25^ This approach trains a BA model based on shared biomarkers measured in the US Health and Nutrition Examination Survey III (NHANES III), a nationally representative sample of 9926 individuals. In total, 17 biomarkers were collected in both CCMR One and NHANES III. Using criteria previously applied for Klemera–Doubal method biomarker selection,^18^ five biomarkers were excluded due to weak correlation with CA (| *r* | < 0.1) and two excluded due to non-independence with other biomarkers (| *r* | > 0.4). The final panel of 10 biomarkers comprised 9 laboratory blood tests (mean cell volume, red cell distribution width, red blood cell count, lymphocyte percentage, urea, albumin, alkaline phosphatase, total cholesterol and triglycerides) as well as systolic blood pressure. Any missing data (Supplementary Table 2) were imputed using the *mice* package with predictive mean matching.^26^

To make BA_Blood_ comparable with BA_MRI_ (which is naïve to CA), the *BioAge* code was modified such that CA was *not* included as a biomarker for the KDM algorithm.^17,27^ Similarly, a single model was applied for both males and females, consistent with the approach taken for MRI brain age. Because all participants of CCMR One were taking dimethyl fumarate, which is known to reduce lymphocyte counts, results were verified using a separate model that did not include lymphocyte percentage as a biomarker. The BA_Blood_ measures (both with and without lymphocyte percentage) were validated as BA measures in the independent NHANES IV testing set and showed statistically significant associations with all-cause mortality and age-related functional outcomes adjusted for CA (Supplementary Table 3).^25^

##### Statistical analysis

Values for BA advancement (a term sometimes referred to in the brain age literature as predicted age difference or brain age gap) were calculated by subtracting CA from raw BA: a positive BA_MRI_ advancement or BA_Blood_ advancement therefore indicates that the individual’s BA is predicted to be older than their CA. Linear models were used to test the effect of bexarotene treatment on BA advancement and the association between baseline BA advancement and remyelination outcomes, with full model details provided in the Supplementary Methods. Magnetization transfer ratio analysis was performed using lesion-level data nested with participants, and VEP analysis was performed using eye-level data nested within participants. Residuals of all models were examined for departures from normality and homoscedasticity. Where assumptions were not met, confidence intervals (CIs) were verified using a bootstrap approach with 500 replicates.

## Results

### Characteristics of biological age measures at study baseline

**Figure 1:**
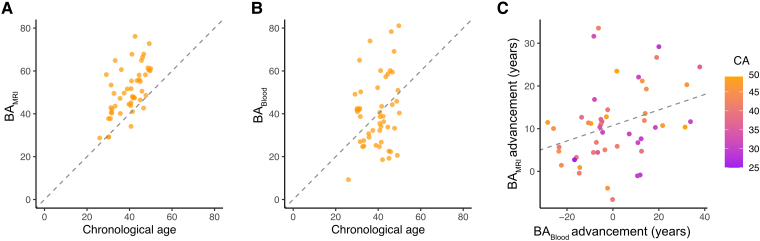
**BA_MRI_ and BA_Blood_ amongst CCMR One participants at study baseline.** (**A**) BA_MRI_ and (**B**) BA_Blood_ are shown plotted against CA for all participants (*n* = 49), with the dashed line representing BA = CA and the vertical distance of a point above this line representing the BA advancement. (**C**) BA_MRI_ advancement plotted against BA_Blood_ advancement, with points coloured based on CA and a dashed line showing the regression line (Pearson *r* = 0.33, *P* = 0.021). BA, biological age; CA, chronological age.

We first characterized the BA measures for the 49 participants at study baseline. BA_MRI_ at baseline was on average 10.9 years higher than CA, with a standard deviation (SD) of 9.0 years (Fig. 1A). In contrast, BA_Blood_ was on average 1.0 years higher than CA (SD 16.3 years; Fig. 1B). Both measures were positively correlated with CA (*r* = 0.60 for BA_MRI_; *r* = 0.22 for BA_Blood_) and with each other (*r* = 0.39). The advancement relative to CA (BA—CA) for BA_MRI_ and BA_Blood_ were also positively correlated with one another (*r* = 0.33), although some outliers had a high BA­_MRI_ advancement with a negative BA_Blood_ advancement (Fig. 1C).

Next, we looked at the relationship between baseline BA advancement and baseline clinical disability as measured using the Expanded Disability Status Scale (EDSS). An increase in BA_MRI_ by 10 years was positively but non-significantly associated with a greater level of disability at baseline after adjustment for CA [0.16 point increase in EDSS, 95% confidence interval (CI) [−0.36, 0.68], *P* = 0.55]. The same increment in blood-based BA was associated with a 0.13 point increase in EDSS (CI [−0.39, 0.65], *P* = 0.38).

### BA_MRI_ is rejuvenated by bexarotene

**Figure 2:**
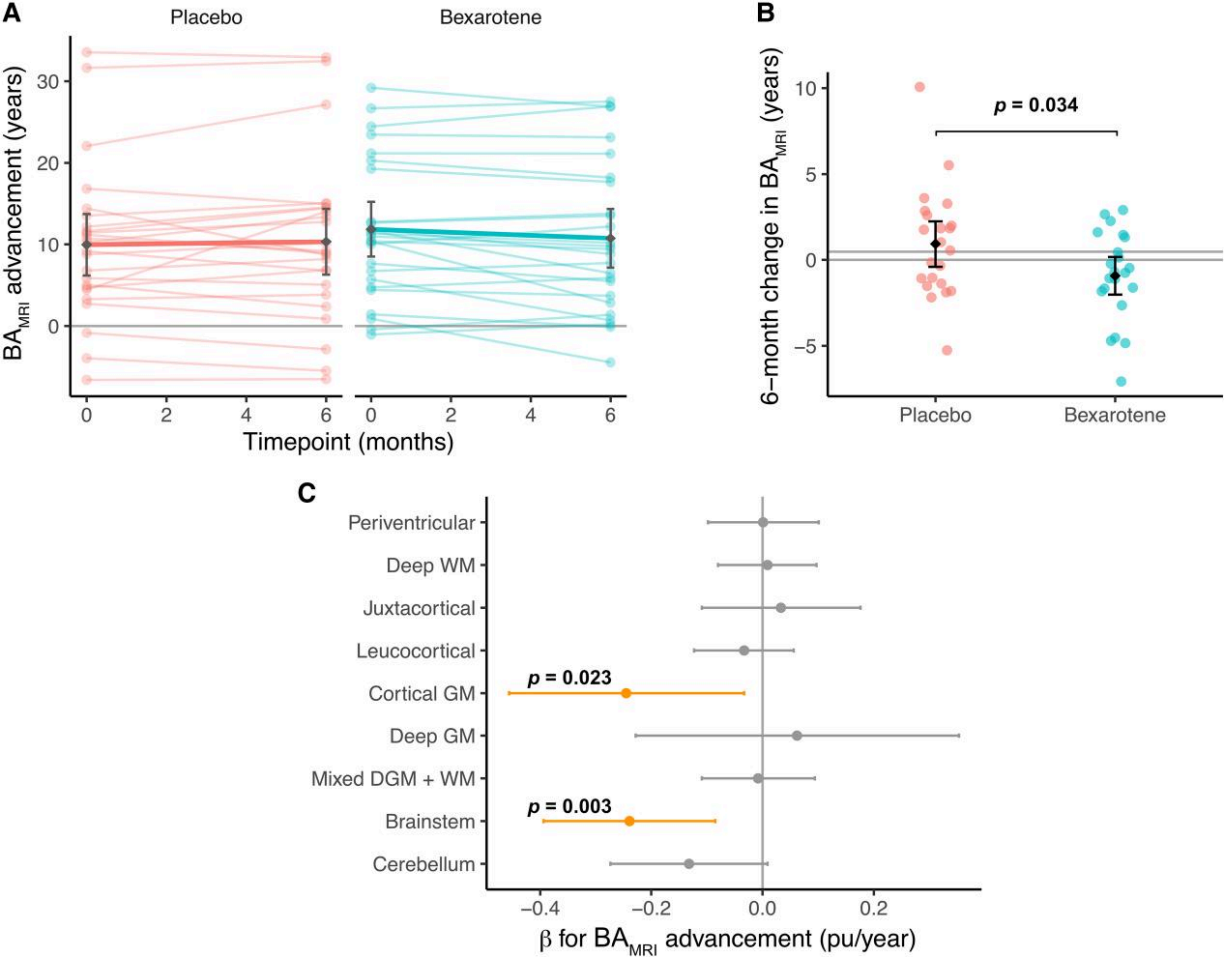
**Effect of bexarotene treatment on BA_MRI_ advancement during the CCMR One trial.** (**A**) Longitudinal change in BA_MRI_ advancement for all participants (*n* = 49). Thin lines show individual trajectories with thick lines denoting the mean change for each group. (**B**) Change in BA_MRI_ after 6 months of treatment compared to baseline in the bexarotene and placebo groups for participants with radiologically stable multiple sclerosis (*n* = 44). The mean and 95% CI are shown with each point representing a participant. The horizontal lines represent 0 (no change in BA_MRI_) and 6 months (the expected change during the trial). Linear model *β* for bexarotene group = −1.98 years (*P* = 0.034). (**C**) Effect size (*β*) and 95% CI by brain region for a marginal change in BA_MRI_ advancement during the trial on remyelination, as assessed by the change in MTR. A shift to the left indicates that a reduction in BA_MRI_ advancement is associated with an increase in lesion MTR (indicating remyelination) for a given region. Regions where this effect is significant (*α* = 0.05) are plotted in orange, with accompanying *P*-values. Linear mixed model using lesions nested within patients: *β* for Δ BA_MRI_ advancement in cortical GM lesions = −0.25 pu/year (*P* = 0.023) and in brainstem lesions *β* = −0.24 pu/year (*P* = 0.003). CI, confidence interval; DGM, deep grey matter; GM, grey matter; MTR, magnetization transfer ratio; WM, white matter.

We next explored how BA_MRI_ changes longitudinally after exposure to bexarotene (Fig. 2A). Amongst those participants with radiologically stable multiple sclerosis (no new lesions seen during the trial, *n* = 44), we found that BA_MRI_ progressed more slowly in the bexarotene group compared to the placebo group (−1.98 years, CI [−3.75, −0.21 years], *P* = 0.034; Fig. 2B). Compared to the start of the trial, BA_MRI_ advancement within the bexarotene group reduced by 1.40 years (CI [0.24, 2.57] years, *P* = 0.020), indicating that after a 6-month drug treatment, participant’s brains appeared on average 11 months younger than they had been at baseline (−0.93 years, CI [−2.02, 0.17], *P* = 0.018 versus expected 0.5-year increase). This was in contrast to the placebo group, in which BA_MRI_ increased as expected during the 6-month treatment period (+0.92 years, CI [−0.41, 2.26], *P* = 0.54).

Effect sizes were of a similar magnitude in sensitivity analysis with inclusion of the five people with new MRI lesions: a −1.53 year difference was seen compared to placebo (CI [−3.14, 0.08] years, *P* = 0.069) and a post-treatment reduction of BA_MRI_ advancement within the bexarotene group of 1.11 years (CI [0.04, 2.18] years, *P* = 0.042).

We next explored factors underlying this apparent rejuvenation of MRI brain age. At the participant level, there were no significant associations between demographic features and observed reduction in BA_MRI_. Individuals with the greatest reduction in BA_MRI_ during bexarotene treatment (>1 SD, *n* = 5) were all female (*χ*^2^, *P* = 0.13) but spanned a relatively wide range of CA (33–47) and EDSS (1.0–6.0). Using lesion-level data nested within participants, we then modelled how remyelination in different brain regions, as assessed by an increase in lesion MTR, was associated with the longitudinal change in BA_MRI_. A reduction in BA_MRI_ advancement during the trial was significantly associated with a greater rise in MTR (reflecting greater remyelination) in CGM lesions (*β* = 0.25% units (pu)/year, CI [0.03, 0.46], *P* = 0.023) and brainstem lesions (*β* = 0.24 pu/year, CI [0.09, 0.39], *P* = 0.003), but not in lesions within other brain regions (Fig. 2C).

To investigate whether this association might represent changes in more diffuse patterns of myelination, we next analysed MTR in CGM and brainstem outside lesion areas (Supplementary Fig. 1). There was no significant difference in non-lesion MTR in the bexarotene versus placebo group in either CGM (−0.04 pu [CI: −0.43, 0.34], *P* = 0.84) or brainstem (+0.56 pu [CI: −0.29, 1.40], *P* = 0.20). Additionally, we compared global volume changes in GM and WM between the two groups (Supplementary Fig. 2). Whilst there was a slight trend towards GM preservation in the bexarotene group—consistent with the beneficial effect on BA_MRI_—this was not statistically significant (+247 mm^3^ [CI: −3343, 3838], *P* = 0.89). There was similarly no significant difference in WM volume change between the groups (−234.56 mm^3^ [CI: −3626, 3157], *P* = 0.89).

We also assessed how BA_Blood_ changed with bexarotene treatment. In contrast to BA_MRI_, BA_Blood_ advancement increased in the treatment arm versus placebo (+10.62 years, CI [1.79, 19.45], *P* = 0.026, Supplementary Fig. 3). Although several individual blood biomarkers were perturbed by bexarotene treatment (Supplementary Table 4), the change in BA_Blood_ was largely explained by hypertriglyceridaemia—a side effect experienced by almost all participants in the treatment arm^16^—and it was attenuated by adjustment for triglyceride concentrations (+3.62 years, CI [−4.49, 11.73], *P* = 0.39). BA_Blood_ advancement returned to baseline levels 3 months after treatment cessation, corresponding to the normalization of these blood biomarkers (Supplementary Fig. 3).

### Biological age measures and visual-evoked potential remyelination outcomes

Based on our previous work that demonstrated attenuation of bexarotene’s remyelinating effects with increasing CA,^7^ we next investigated whether baseline BA_MRI_ and BA_Blood_ would have similar effects to CA on remyelination outcomes. We first studied the effect of these measures on VEP latency reduction, which is an electrophysiological biomarker for remyelination within the visual system.

**Figure 3:**
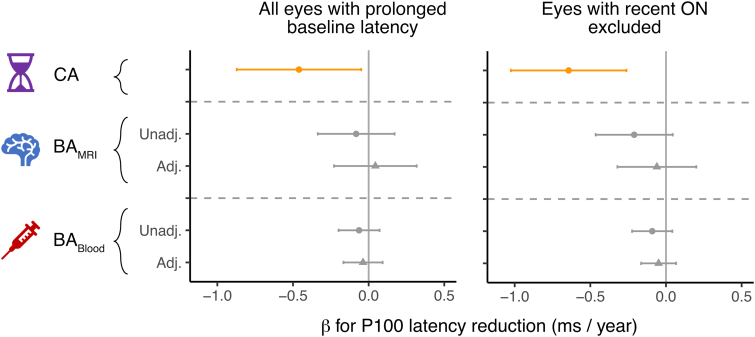
**Association of CA and BA measures at baseline with remyelination in response to bexarotene, assessed using visual evoked potentials.** Effect size (*β*) and 95% CI for a marginal increase in CA, BA_MRI_ or BA_Blood_ on the reduction in P100 latency response. Data are shown for all eyes with prolonged latency at baseline (*n* = 51 eyes from 28 participants) and separately after exclusion of eyes that have a recent history of ON (*n* = 42 eyes from 26 participants). In the unadjusted models (circle points), the BA measure replaces CA in the model, whereas the adjusted models (triangular points) are simultaneously adjusted for the effect of CA. BA, biological age; CA, chronological age; ON, optic neuritis.

The VEP latency reduction from bexarotene treatment was reduced by 0.46 ms (CI [0.05, 0.87], *P* = 0.035) for every year increase in CA. When BA_MRI_ was substituted into the model in place of CA, a downward trend in VEP response was also apparent but with a smaller magnitude of effect (0.08 ms/year, CI [−0.17, 0.34], *P* = 0.53; Fig. 3; Supplementary Table 5). This increased to 0.21 ms/year (CI [−0.04, 0.46], *P* = 0.12) when excluding the eyes that had suffered from optic neuritis more recently than 5 years preceding the trial, in order to minimize noise in the longitudinal VEP assessments (*n* = 42 eyes, following exclusion of nine eyes). When BA_Blood_ was substituted into the model, the associated decrement in VEP response was non-significant. In particular, after adjusting for CA in the models alongside BA measures, the effect sizes of an increase in BA_MRI_ or BA_Blood_ advancement for a given CA were generally close to 0, albeit magnified by the exclusion of those eyes with recent optic neuritis (Fig. 3; Supplementary Table 5). These two measures of BA therefore did not explain the variability in remyelination (as assessed by VEP) significantly beyond the effect of CA.

#### Biological age measures and MRI remyelination outcomes

**Figure 4:**
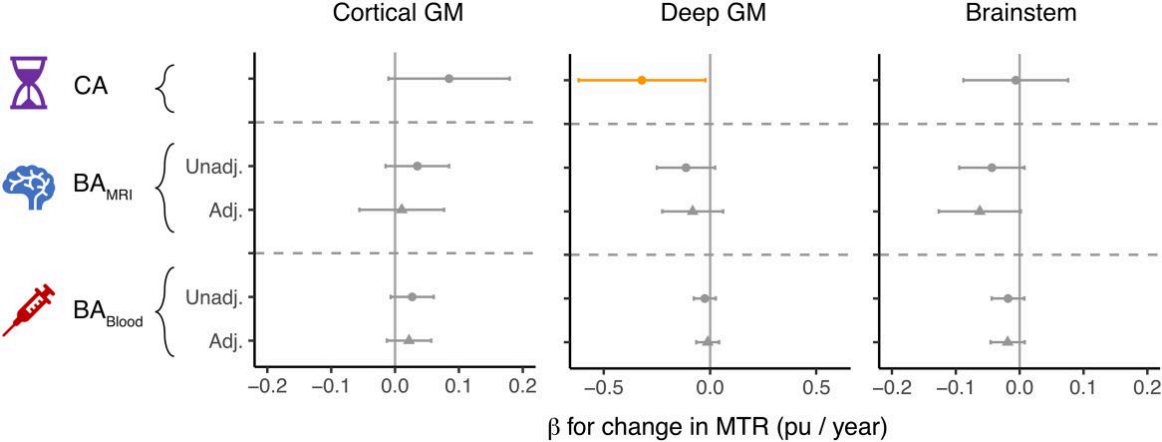
**Association of CA and BA measures at baseline with remyelination in response to bexarotene, assessed using lesion MTR.** Effect size (*β*) and 95% CI for a marginal increase in CA, BA_MRI_ or BA_Blood_ on the change in lesion MTR signal (*n* = 49 participants). In the unadjusted models (circular points), the BA measure replaces CA in the model, whereas the adjusted models (triangular points) are simultaneously adjusted for the effect of CA. BA, biological age; CA, chronological age; GM, grey matter; MTR, magnetization transfer ratio.

We next modelled the effect of BA measures at baseline on the change in lesion MTR, focusing on the regions most affected by bexarotene.^16^ Whilst increasing CA attenuated the MTR response in DGM by 0.32 pu/year (CI [0.02, 0.62], *P* = 0.035; Fig. 4; Supplementary Table 6), this effect was smaller for increments in BA_MRI_ (0.11 pu/year, CI [−0.02, 0.25], *P* = 0.10) and further reduced when also adjusting for CA (0.08 pu/year, CI [−0.06, 0.22], *P* = 0.26). BA_Blood_ had little effect on DGM remyelination (reduction by 0.03 pu/year, CI [−0.03, 0.08], *P* = 0.34).

In contrast, although CA had no effect on brainstem remyelination in response to bexarotene (reduction by 0.01 pu/year, CI [−0.08, 0.09], *P* = 0.88), increasing baseline BA_MRI_ was associated with a borderline significant decrement in brainstem remyelination (reduction by 0.06 pu/year in adjusted model, CI [0.00, 0.13], *P* = 0.058). A negative trend was also seen for BA_Blood_ in brainstem remyelination (reduction by 0.02 pu/year in adjusted model, CI [−0.01, 0.05], *P* = 0.17).

Unlike the other regions studied, CGM remyelination in response to bexarotene was well maintained at older CA, and in fact, there was some signal that older CA was associated with more effective CGM remyelination (increase by 0.08 pu/year, CI [−0.01, 0.18], *P* = 0.081). The association of CGM remyelination with BA_MRI_ or BA_Blood_ was weaker than for CA (increase by 0.01 pu/year, CI [−0.06, 0.08], *P* = 0.76, and 0.02 pu/year, CI [−0.01, 0.06], *P* = 0.22, respectively, in adjusted models) and not statistically significant.

Given that all participants were concurrently taking dimethyl fumarate, which is known to cause lymphopaenia, the VEP and MTR remyelination outcomes were reanalysed using a version of BA_Blood_ that does not include lymphocyte percentage as a biomarker. At baseline, this measure was on average 1.7 years younger than expected for CA (SD 16.1 years) compared to 1.0 years older (SD 16.3 years) for the measure incorporating lymphocytes. The effects on remyelination outcomes were largely consistent with those calculated using the lymphocyte-inclusive BA_Blood_ (Supplementary Tables 7 and 8).

## Discussion

Here, we report evidence supporting a relationship between measures of biological ageing and remyelination in people with multiple sclerosis. We see a slowing of brain ageing, indeed apparent rejuvenation (a fall in BA_MRI_), for participants receiving bexarotene, a drug that promotes remyelination. To our knowledge, this is the first demonstration that MRI brain age of people with a neurological disorder can be therapeutically modified in response to a drug. Although one study in healthy young adults (*n* = 20) found a significant effect of ibuprofen treatment,^28^ the notion that MRI brain age might respond to interventions in adult life has previously been questioned.^29^ People with multiple sclerosis display accelerated brain age compared to healthy controls, and higher brain age advancement is associated with greater disability.^11^ One interpretation of our results is that during remyelination, we see a reversal or slowing of some of the radiological features of multiple sclerosis that give rise to an older-appearing brain. This is supported by the finding that remyelination in two regions that are particularly sensitive to bexarotene (cortical grey matter and brainstem; based on regional therapeutic response of lesional MTR in CCMR One^16^) is significantly associated with this reduction in brain age advancement. However, we cannot discount that some artefactual changes with bexarotene, for example, oedema, may contribute to the observed effect. Notably, our blood-based BA measure was susceptible to hypertriglyceridaemia as a side effect of bexarotene treatment. This may be artefactual, or a genuine (although reversible) increase in BA_Blood_, given that sustained hypertriglyceridaemia over time leads to accelerated age-related cardiovascular morbidity.^30^

This work also builds on the previously described effects of CA on bexarotene-induced remyelination,^7^ by testing the association between two BA measures at study baseline and subsequent remyelination. After full adjustment for CA, a higher BA_MRI_ or BA_Blood_ tended to have a negative effect, suggesting that being biologically older than one’s CA has an adverse effect on remyelination in people receiving bexarotene. However, in this clinical cohort of 49 people, effect sizes were small and generally not statistically significant. For example, given the same CA and no recent history of optic neuritis, a 1 SD increase in BA_MRI_ (+9.0 years) or BA_Blood_ (+16.3 years) would be expected to shrink the P100 latency improvement by 0.5 ms or 0.8 ms, respectively. For reference, the total treatment-related effect for those without recent optic neuritis in CCMR One was an improvement of 4.75 ms (CI [0.71, 8.80]).^16^ Interestingly, the strongest effect we observed here is that an older-appearing baseline BA_MRI_ brain age is associated with poor brainstem remyelination, which in turn is associated with less brain age rejuvenation.

There are several examples in the literature of BA measures being applied to cohorts of people with multiple sclerosis. One study in California (*n* = 516) showed that a shorter telomere length at baseline predicted future disability accumulation despite adjustment for CA.^31^ A Swedish study (*n* = 320) calculated four methylation-based age measures and found one (but not others) to be accelerated in people with multiple sclerosis compared to healthy controls.^32^ However, BA has not been previously related to measures of remyelination in a clinical cohort.

This study made use of existing data from a clinical trial, which allows comparison between double-blinded treatment and placebo arms, but also brings limitations. For example, we can only calculate BA measures using the data that was collected contemporaneously—namely, safety-monitoring blood tests and MRI scans—and we are unable to use other approaches such as epigenetic clocks or telomere length. On the other hand, the data used here are routinely collected in clinical practice, making these measures of BA directly translatable. A further limitation of using this data *post hoc* is the use of FLAIR to detect cortical lesions. This approach is considerably less sensitive than dedicated cortical lesion sequences and does not allow the identification of intracortical or purely subpial lesions, which limit that generalizability of our findings to other CGM lesions.^33^ The modest sample size (*n* = 49) compared to previous cohort studies of BA also brings a risk of type II error, which we have attempted to mitigate through sensitivity analysis excluding those with less quiescent disease (recent optic neuritis or MRI activity) to improve signal to noise.

In conclusion, we found that treatment of people with RRMS with bexarotene to promote remyelination is associated with a rejuvenation effect on BA_MRI_. This effect was principally related to remyelination in cortical grey matter and brainstem lesions. We also observe trends that an older BA_MRI_ or BA_Blood_ relative to CA at baseline is associated with less capacity for therapeutic remyelination, although CA has a more dominant effect.

## Supplementary Material

fcaf032_Supplementary_Data

## Data Availability

The following trial data sets (including data dictionaries) are available for researchers from our website: de-identified participant data, the primary efficacy endpoint data set, the visual evoked potentials data set and the lesional magnetization transfer ratio data set. Access requests should be made to the chief investigator (A.J.C.; ajc1020@medschl.cam.ac.uk). A signed data access agreement will be required and investigator support might be provided if part of an academic collaboration.
