# Supplementary material for: Exploratory analysis of biological age measures in a remyelination clinical trial
Source: Brain Commun. 2025 Jan 29;7(1):fcaf032. doi: 10.1093/braincomms/fcaf032 (PMC11852336; doi:10.1093/braincomms/fcaf032)
Supplement: fcaf032_Supplementary_Data [file fcaf032_Supplementary_Data.pdf]

Supplementary Methods – statistical models

A two-sided paired *t*-test was used to compare pre- and post-treatment BA_{MRI} advancement within the bexarotene group.

The effect of treatment on change in BA_{MRI} advancement (Δ BA_{MRI} adv) was tested using a linear model with adjustment for baseline BA_{MRI} advancement to account for any initial asymmetry between the bexarotene and placebo groups:

$$\Delta\ BA_{MRI}\ adv \sim treatment + baseline\ BA_{MRI}\ adv \qquad (1)$$

The association between change in BA_{MRI} advancement and regional MTR change within lesions (Δ MTR_{Lesion}) was tested using lesion-level data, in a linear mixed model for lesions nested within participants:

$$\Delta\ MTR_{Lesion} \sim\ \Delta\ BA_{MRI}\ adv * brain\ region + baseline\ BA_{MRI} + baseline\ MTR_{Lesion} + treatment + (\ 1\ |\ participant) \qquad (2)$$

The effect of treatment on change in non-lesion MTR (Δ $MTR_{Non\text{-}lesion}$) in cortical grey matter and in brainstem was calculated using an equivalent model to (1):

$$\Delta\ MTR_{Non-lesion} \sim treatment + baseline\ MTR_{Non-lesion} \qquad (3)$$

The effect of treatment on volume changes (Δ Volume) in grey matter (combined cortical and deep grey matter) and in white matter was assessed using a similar model, with additional adjustment for baseline total intracranial volume (TIV):

$$\Delta\ Volume \sim treatment + baseline\ volume + TIV \qquad (4)$$

Models to test the effect of baseline BA advancement on remyelination outcomes were based on those used for the original trial endpoints and post-hoc analysis of CA in CCMR One.[1,2] Visual evoked potential analysis used linear mixed models for eyes nested within participants. Firstly, the effect of CA was modelled, by regressing the change in P100 latency on an interaction between CA, treatment group, and baseline value (≤118/ >118 ms), as well as the trial minimisation factors gender and trial centre:

$$\Delta\ P100\ latency\ \sim\ CA * treatment * baseline\ P100\ latency + gender + centre + (\ 1\ |\ participant) \qquad (5)$$

To assess the effect of BA, a raw BA term (BA_{MRI} or BA_{Blood}) was substituted in place of CA in this model ("basic model"):

$$\Delta\, P100\, latency \sim BA * treatment * baseline\, P100\, latency + gender + centre + (\, 1 \,|\, participant) \quad (6)$$

To assess the marginal effect of BA_{MRI} or BA_{Blood} advancement after adjustment for CA, the advancement term was added to the CA model, in interaction with treatment and baseline P100 ("adjusted model"):

$$\Delta\, P100\, latency \sim CA * treatment * baseline\, P100\, latency + BA\, adv + BA\, adv : treatment + BA\, adv : baseline\, P100\, latency + gender + centre + (\, 1 \,|\, participant) \quad (7)$$

No further adjustment was made for baseline EDSS (also a minimisation factor) as this is known to be closely related to CA.

Similarly for lesion MTR analysis, linear mixed models were used for lesions nested within participants, with the initial CA model as follows:

$$\Delta\, MTR_{Lesion} \sim CA * treatment * brain\, region + baseline\, MTR_{Lesion} + gender + centre + (\, 1 \,|\, participant) \quad (8)$$

The "basic model" was derived by substituting raw BA (BA_{MRI} or BA_{Blood}) for CA, whilst the "adjusted model" was derived by including a BA advancement term in interaction with treatment and brain region.

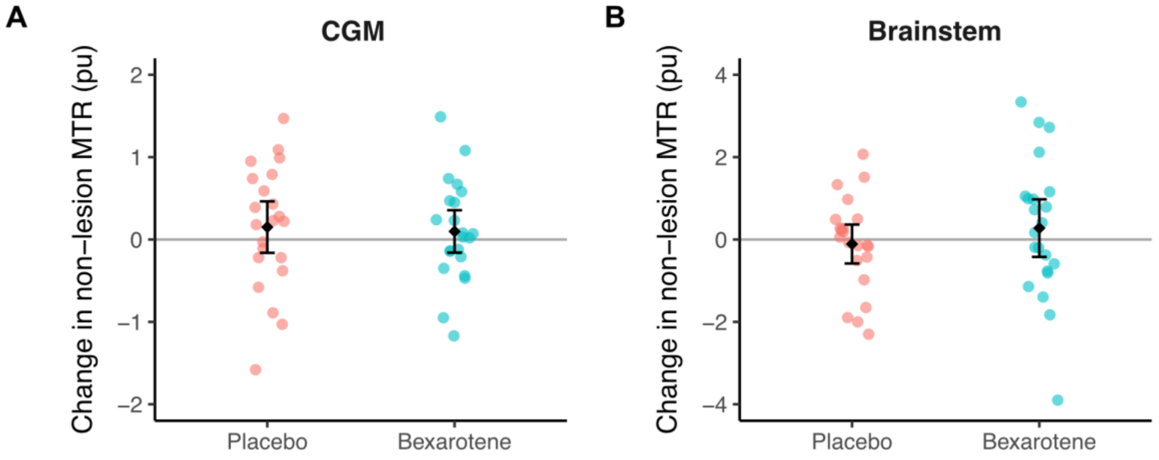

Supplementary Figure 1: Change in MTR in non-lesion areas of CGM and brainstem. The change in mean non-lesion MTR of CGM (**A**) and brainstem (**B**) between baseline and 6 months in the bexarotene and placebo groups, for participants with radiologically stable MS (*n* = 44). The mean and 95% CI are shown with each point representing a participant. CGM = cortical grey matter, MTR = magnetisation transfer ratio.

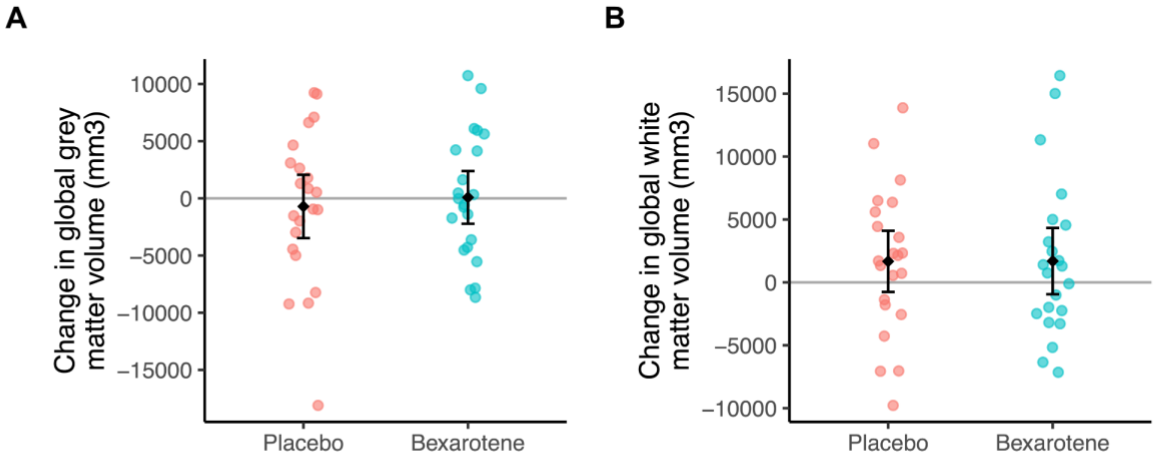

Supplementary Figure 2: Changes in global grey matter and white matter volume. The change in global grey matter volume (combined CGM and DGM, **A**) and global white matter volume (**B**) between baseline and 6 months in the bexarotene and placebo groups, for participants with radiologically stable MS (*n* = 44). This analysis includes both lesion and non-lesion tissue. The mean and 95% CI are shown with each point representing a participant. CGM = cortical grey matter, DGM = deep grey matter.

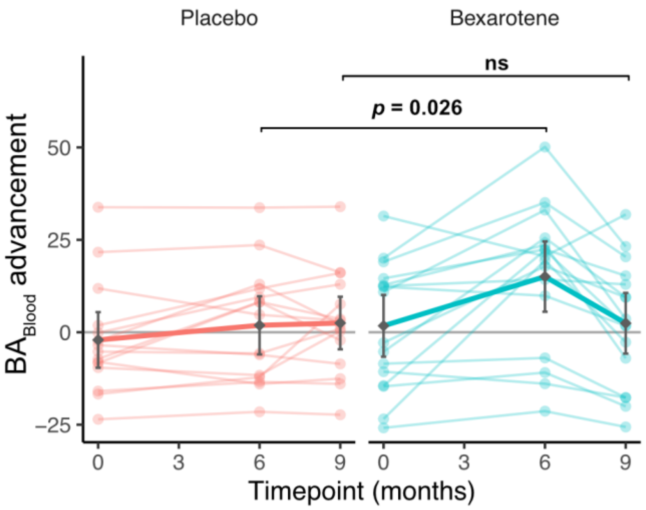

Supplementary Figure 3: Effect of bexarotene treatment on BA_{Blood} advancement during the CCMR One trial. Longitudinal change in BA_{Blood} advancement for all participants (n = 31, Cambridge centre only). Thin lines show individual trajectories with thick lines denoting the mean change for each group. There was an additional timepoint of blood test data at 9 months (unlike in the MRI data; **Figure 2**), which was built into the trial protocol to review whether abnormalities normalised 3 months after treatment cessation. Linear model β for bexarotene group at 6 months = 10.6 (p = 0.026); at 9 months = -2.63 (p = 0.46). BA = biological age; ns = not significant.

Supplementary Table 1: Summary of numbers of patients used in sensitivity analyses.

	Total	Bexarotene	Placebo
Participants in full intention-to-treat cohort	49	25	24
Participants with no new lesions on follow-up MRI	44	22	22
Participants with baseline P100 > 118ms (n_{eyes})	28 (51)	16 (29)	12 (22)
Participants with baseline P100 > 118ms AND no clinical optic neuritis in preceding 5 years (n_{eyes})	26 (42)	15 (25)	11 (17)

Supplementary Table 2: Summary of missing data for blood biomarkers at baseline in the CCMR One trial.

	$n_{missing}$ (%)
Red cell distribution width	18 (36.7%)
Urea	7 (14.2%)
Red blood cell count	1 (2.0%)
Albumin	1 (2.0%)
Systolic blood pressure	1 (2.0%)

Supplementary Table 3: Associations of the blood-based BA measure with mortality and other age-associated health outcomes. CA-adjusted advancement in the original KDM BA measure (Levine 2013)[3] and in our version of blood-based BA (which uses the biomarkers available within CCMR One and is naïve to CA) were tested using outcome data from NHANES IV. ADLs = activities of daily living; BA = biological age; KDM = Klemera-Doubal method.

	**CCMR One blood-based BA advancement**	**CCMR One blood-based BA advancement** (without lymphocyte %)	**Original BA_{KDM} advancement** (Levine 2013)[3]
**Mortality**			
Hazard ratio (95% CI)	1.28 (1.22, 1.34)	1.26 (1.20, 1.32)	1.36 (1.20, 1.55)
**Age-associated health outcomes**			
β (95% CI)			
Self-rated health[1]	0.15 (0.14, 0.17)	0.15 (0.14, 0.17)	0.25 (0.23, 0.27)
Impairment in ADLs[2]	0.08 (0.06, 0.10)	0.07 (0.05, 0.09)	0.13 (0.10, 0.16)
Time to complete 20-foot walk (s)[3]	0.07 (0.03, 0.10)	0.06 (0.03, 0.10)	-
Grip strength (kg)	-0.02 (-0.04, 0.01)	-0.01 (-0.04, 0.02)	-

[1] 5-point scale from 1 (excellent) to 5 (poor)
[2] NHANES ADL instrument, in which higher values indicate more difficulty with activities
[3] Log-transformed

Supplementary Table 4: Changes in individual blood-based biomarkers during bexarotene treatment. Data is shown for the *n* = 31 Cambridge-based participants with longitudinal data available.

**Biomarker**[1]	**Change during 6-month treatment period**	***p*-value**
Mean cell volume (fL)	0.47	0.59
Red cell distribution width (%)	0.16	0.60
**Lymphocyte percentage (%)**	**10.57**	**1.1×10^{-3}**
**Albumin (g/L)**	**- 2.84**	**1.9×10^{-4}**
Alkaline phosphatase (U/L)	- 6.26	0.35
**Red blood cell count (10^{12}/L)**	**- 0.33**	**3.1×10^{-3}**
**Triglycerides (mmol/L)**	**1.21**	**7.1×10^{-3}**
**Total cholesterol (mmol/L)**	**1.42**	**2.3×10^{-3}**
Systolic blood pressure (mmHg)	- 1.62	0.68

[1] NB urea was not measured longitudinally

Supplementary Table 5: Association of chronological age and biological age measures at baseline with remyelination in response to bexarotene, assessed using P100 latency reduction.

	**Effect on VEP latency reduction**	
	β (95% CI); ms/year	*p*-value
**Chronological age**		
All eyes	-0.46 (-0.87, -0.05)	0.035
ON excluded	-0.64 (-1.03, -0.26)	0.003
**MRI brain age**		
All eyes	-0.08 (-0.34, 0.17)	0.53
ON excluded	-0.21 (-0.46, 0.04)	0.12
Adjusted model; all eyes	0.04 (-0.23, 0.32)	0.75
Adjusted model; ON excluded	-0.06 (-0.32, 0.20)	0.65
**Blood-based biological age**		
All eyes	-0.06 (-0.20, 0.07)	0.37
ON excluded	-0.09 (-0.22, 0.04)	0.19
Adjusted model; all eyes	-0.04 (-0.17, 0.09)	0.58
Adjusted model; ON excluded	-0.05 (-0.16, 0.07)	0.42

Supplementary Table 6: Association of chronological age and biological age measures at baseline with remyelination in response to bexarotene, assessed using change in lesion magnetisation transfer ratio (ΔMTR).

	Effect on CGM ΔMTR		Effect on DGM ΔMTR		Effect on brainstem ΔMTR	
	β (95% CI); pu/year	*p*-value	β (95% CI); pu/year	*p*-value	β (95% CI); pu/year	*p*-value
**Chronological age**	0.08 (-0.01, 0.18)	0.081	-0.32 (-0.62, -0.02)	0.035	-0.01 (-0.09, 0.08)	0.88
**MRI brain age**						
Unadjusted model	0.04 (-0.01, 0.08)	0.17	-0.11 (-0.25, 0.02)	0.10	-0.04 (-0.09, 0.01)	0.096
Adjusted model	0.01 (-0.06, 0.08)	0.76	-0.08 (-0.22, 0.06)	0.26	-0.06 (-0.13, 0.00)	0.058
**Blood-based biological age**						
Unadjusted model	0.03 (-0.01, 0.06)	0.12	-0.03 (-0.08, 0.03)	0.34	-0.02 (-0.04, 0.01)	0.16
Adjusted model	0.02 (-0.01, 0.06)	0.22	-0.01 (-0.06, 0.04)	0.68	-0.02 (-0.05, 0.01)	0.17

Supplementary Table 7: Association of blood-based biological age – excluding lymphocyte percentage as a biomarker – at baseline with remyelination in response to bexarotene, assessed using P100 latency reduction.

	**Effect on VEP latency reduction**	
	β (95% CI); ms/year	*p*-value
**Blood-based biological age (without lymphocyte %)**		
All eyes	-0.05 (-0.19, 0.08)	0.45
ON excluded	-0.08 (-0.21, 0.06)	0.26
Adjusted model; all eyes	-0.03 (-0.16, 0.10)	0.67
Adjusted model; ON excluded	-0.04 (-0.15, 0.08)	0.51

Supplementary Table 8: Association of blood-based biological age – excluding lymphocyte percentage as a biomarker – at baseline with remyelination in response to bexarotene, assessed using change in lesion magnetisation transfer ratio (ΔMTR).

	**Effect on CGM ΔMTR**		**Effect on DGM ΔMTR**		**Effect on brainstem ΔMTR**	
	β (95% CI); pu/year	*p*-value	β (95% CI); pu/year	*p*-value	β (95% CI); pu/year	*p*-value
**Blood-based biological age without lymphocyte %**						
Unadjusted model	0.03 (-0.00, 0.06)	0.09	-0.03 (-0.08, 0.03)	0.33	-0.02 (-0.04, 0.01)	0.16
Adjusted model	0.03 (-0.01, 0.06)	0.15	-0.01 (-0.07, 0.04)	0.66	-0.02 (-0.05, 0.01)	0.16

Supplementary References

1. McMurran CE, Mukherjee T, Brown JWL, et al. Remyelination in humans due to a retinoid-X receptor agonist is age-dependent. Ann Clin Transl Neurol 2022;9:1090–1094.

2. Brown JWL, Cunniffe NG, Prados F, et al. Safety and efficacy of bexarotene in patients with relapsing-remitting multiple sclerosis (CCMR One): a randomised, double-blind, placebo-controlled, parallel-group, phase 2a study. Lancet Neurol 2021;20:709–720.

3. Levine ME. Modeling the rate of senescence: Can estimated biological age predict mortality more accurately than chronological age? Journals of Gerontology - Series A Biological Sciences and Medical Sciences 2013;68:667–674.

Supplementary Appendix – *R* code used to generate figures and statistics

Setup

```
library(psych)
library(lme4)
library(lmerTest)
library(dplyr)
library(ggplot2)
library(ggeffects)
library(tidyr)
library(RColorBrewer)
library(reshape2)
library(BioAge)
library(flexsurv)
library(stringr)

#Load datasets
load("DataSets/DataSetsForPaper/brainage_all_clean.Rda") #Bloods
load("DataSets/DataSetsForPaper/all_bloods_table_long.Rda") #Brainages

#Trial outcome datasets:
load("DataSets/DataSetsForPaper/bline_ITT_clean.Rda")
load("DataSets/DataSetsForPaper/bline_long_eyes_ITT_clean.Rda")
load("DataSets/DataSetsForPaper/bline_ITT_bypatient_clean.Rda")

#Rename
brainage_all <- brainage_all_clean

bline_ITT_bypatient <- bline_ITT_bypatient_clean
bline_long_eyes_ITT <- bline_long_eyes_ITT_clean
bline_ITT <- bline_ITT_clean

treatment.labs <- c("Placebo", "Bexarotene")
```

1. Brain age processing

```
#Make long dataframe for all timepoints
brainage_all_long <- brainage_all %>% reshape(direction = "long",
                         varying=c("age.0","age.6",
                                   "brainage.0", "brainage.6",
                                   "brainage_advance.0", "brainage_advance.6",
                                   "CGMvol.0", "CGMvol.6",
                                   "DGMvol.0", "DGMvol.6",
                                   "brainstemvol.0", "brainstemvol.6",
                                   "TIV.0", "TIV.6",
                                   "CGMmtr.0", "CGMmtr.6",
                                   "DGMmtr.0", "DGMmtr.6",
                                   "brainstemmtr.0", "brainstemmtr.6"),
                         timevar="timepoint",
                         idvar="patient_id")

rownames(brainage_all_long) <- NULL


# Add brainage to trial outcomes datasets

bline_ITT <- bline_ITT %>% merge((brainage_all[,c("patient_id", "brainage.0", "brainage_advance.0", "brainage_advance.change")] %>%
                                  dplyr::rename("brainage" = "brainage.0",
                                                "brainage_advance" = "brainage_advance.0")), by="patient_id")

bline_long_eyes_ITT <- bline_long_eyes_ITT %>% merge((brainage_all[,c("patient_id", "brainage.0", "brainage_advance.0", "brainage_advanc
e.change")] %>%
                                                      dplyr::rename("brainage" = "brainage.0",
                                                                    "brainage_advance" = "brainage_advance.0")), by="patient_id")

bline_ITT_bypatient <- bline_ITT_bypatient %>% merge((brainage_all[,c("patient_id", "brainage.0", "brainage_advance.0", "brainage_advanc
e.change")] %>%
                                                      dplyr::rename("brainage" = "brainage.0",
                                                                    "brainage_advance" = "brainage_advance.0")), by="patient_id")
```

2. Blood age processing

```
#Select KDM variables (correlate with CA r > 0.1)
blood_BMs_full_clinic <- c("mcv",  "rdw", "lymph", "wbc", "albumin_gL", "creat_umol", "alp", "ttbl", "monopa", "rbc", "hdl", "trig", "to
tchol", "bun", "sbp", "dbp", "bmi")
#N.B. Leave out pulse as suspicious distribution in NHANES III

nhanes_cor <- data.frame("biomarker" = NA, "r" = NA, "select" = "")
for (i in 1:length (blood_BMs_full_clinic)){
  variable <- blood_BMs_full_clinic[i]
  nhanes_cor[i,"biomarker"] <- variable
  nhanes_cor[i,"r"] <- cor(NHANES3$age, NHANES3[,variable], use = "pairwise.complete.obs")[1]
}
nhanes_cor$select <- abs(nhanes_cor$r) > 0.1
nhanes_cor

blood_BMs_correlated_clinic <- blood_BMs_full_clinic[nhanes_cor$select]
```

```r
#Note KDM methods optimised for uncorrelated biomarkers; drop one of each pair where |r| > 0.4 (Levine 2012 cutoff)
psych::corPlot(cor(NHANES3[,blood_BMs_correlated_clinic], use = "pairwise.complete.obs"), diag=FALSE, upper=FALSE, min.length=3)
```

```
## Error in plot.new(): figure margins too large
```

```r
# - creat and bun r = 0.53: keep bun (rCA = 0.46), drop creat (rCA = 0.30)
# - sbp and dbp r = 0.49: keep sbp (rCA = 0.59), drop creat (rCA = 0.11)

blood_BMs_final_clinic_reduced <- blood_BMs_correlated_clinic[!blood_BMs_correlated_clinic %in% c("creat_umol", "dbp")]
blood_BMs_final_clinic_reduced

#Modify BioAge package KDM functions to exclude CA, and not split by sex - for more consistency with brainage methods
kdm_calc_no_CA = function (data, biomarkers, fit = NULL, s_ba2 = NULL) {

  dat = data
  bm = biomarkers
  bm_dat = t(select(dat, bm))

  rm(biomarkers)

  if (is.null(fit)) {

    lm_age = t(apply(bm_dat, 1, function(x) {
      sm = summary(lm(x ~ dat$age))
      return(c(sm$sigma, sm$r.sq, sm$coef[1,1], sm$coef[2,]))

    }))

    colnames(lm_age) <- c("RMSE", "r_squared", "B_intercept", "B_age", "se(B)_age", "T_val_age", "pval_age")
    lm_age <- as.data.frame(lm_age)

    lm_age$r1 = abs((lm_age$B_age / lm_age$RMSE) * sqrt(lm_age$r_squared))
    lm_age$r2 = abs((lm_age$B_age / lm_age$RMSE))
    lm_age$n2 = (lm_age$B_age / lm_age$RMSE) * (lm_age$B_age / lm_age$RMSE)

    age_range = range(dat$age, na.rm = TRUE)
    rchar = sum(lm_age$r1) / sum(lm_age$r2)
    s_r = ((1 - (rchar * rchar)) / (rchar * rchar) * (((age_range[2] - age_range[1]) ^ 2) / (nrow(lm_age) * 12)))

  }

  else {

    lm_age = fit$lm_age
    s_r = fit$s_r

  }
  #end dat conditional

  n1 = bm_dat
  for (r in 1:nrow(n1)) {

    x = rownames(n1)[r]
    n1[r,] = ((bm_dat[x,] - lm_age[x,"B_intercept"]) * (lm_age[x,"B_age"] / (lm_age[x,"RMSE"] * lm_age[x,"RMSE"])))

  }

  rm(r); rm(x)

  BAe_n = apply(n1, 2, sum); rm(n1)
  BAe_d = sum(lm_age$n2)

  BAe = BAe_n / BAe_d
  BA_CA = BAe - dat$age

  s2 = sd(BA_CA, na.rm = TRUE) ^ 2
  nobs = sum(!is.na(BA_CA))

  if (is.null(s_ba2)) {

    s_ba2 = s2 - s_r

  }

  else {

    s_ba2 = s_ba2

  }

  dat$kdm2 = (BAe_n) / (BAe_d)
  dat$kdm2_advance = dat$kdm2 - dat$age

  fit = list(lm_age = lm_age, s_r = s_r, s_ba2 = s_ba2, s2 = s2, nobs = nobs)

  kdm2 = list(data = as.data.frame(dat), fit = fit)
  class(kdm2) = append(class(kdm2), "kdm2")
  return(kdm2)

}



kdm_nhanes_no_CA = function (biomarkers) {


  #version without splitting by sex:
```

```r
  train_both = kdm_calc_no_CA(data = NHANES3 %>%
                                filter(age >= 30 & age <= 75 & pregnant == 0),
                                biomarkers, fit = NULL, s_ba2 = NULL)

  test = kdm_calc_no_CA(data = NHANES4, biomarkers, fit = train_both$fit, s_ba2 = train_both$fit$s_ba2)$data


  # #calculate training KDM
  # train_fem = kdm_calc_no_CA(data = NHANES3 %>%
  #                         filter(age >= 30 & age <= 75 & pregnant == 0, gender == 2),
  #                       biomarkers, fit = NULL, s_ba2 = NULL)
  # train_male = kdm_calc_no_CA(data = NHANES3 %>%
  #                          filter(age >= 30 & age <= 75 & pregnant == 0, gender == 1),
  #                        biomarkers, fit = NULL, s_ba2 = NULL)
  #
  # #calculate test modified KDM
  # test_fem = kdm_calc_no_CA(data = NHANES4 %>%
  #                        filter(gender == 2),
  #                      biomarkers, fit = train_fem$fit, s_ba2 = train_fem$fit$s_ba2)
  # test_male = kdm_calc_no_CA(data = NHANES4 %>%
  #                         filter(gender == 1),
  #                       biomarkers, fit = train_male$fit, s_ba2 = train_male$fit$s_ba2)

  #comebine calculated kdm
  # test = rbind(test_fem$data, test_male$data)

  #combine data
  dat = left_join(NHANES4, test[,c("sampleID", "kdm2", "kdm2_advance")], by = "sampleID")
  # fit = list(female = train_fem$fit, male = train_male$fit, nobs = test_fem$fit$nobs + test_male$fit$nobs)
  fit = list(both = train_both$fit, nobs = train_both$fit$nobs)


  kdm2 = list(data = dat, fit = fit)
  class(kdm2) = append(class(kdm2), "kdm2")
  return(kdm2)

}

#Version of KDM without CA in the model
kdmage_extended_fit_no_CA <- kdm_nhanes_no_CA(biomarkers = blood_BMs_final_clinic_reduced)$fit

kdm_CCMROne_noCA <- kdm_calc_no_CA(data = all_bloods_table_long,
                   biomarkers=blood_BMs_final_clinic_reduced,
                   fit = kdmage_extended_fit_no_CA$both)$data[,c("sampleID", "kdm2", "kdm2_advance")]

#Version of KDM without CA in the model; without lymphocyte %
kdmage_extended_fit_no_CA_no_lymph <- kdm_nhanes_no_CA(biomarkers = blood_BMs_final_clinic_reduced[!blood_BMs_final_clinic_reduced %in%
"lymph"])$fit

kdm_CCMROne_noCA_no_lymph <- kdm_calc_no_CA(data = all_bloods_table_long,
                   biomarkers=blood_BMs_final_clinic_reduced[!blood_BMs_final_clinic_reduced %in% "lymph"],
                   fit = kdmage_extended_fit_no_CA$both)$data[,c("sampleID", "kdm2", "kdm2_advance")] %>%

  dplyr::rename("kdm2_no_lymph" = "kdm2", "kdm2_no_lymph_advance" = "kdm2_advance")

#Pull blood BA data together:
all_bloods_table_long_BAs <- all_bloods_table_long %>%
  merge(kdm_CCMROne_noCA, by="sampleID") %>%
  merge(kdm_CCMROne_noCA_no_lymph, by="sampleID") %>%
  merge(bline_ITT_bypatient[,c("patient_id", "active", "active.switched")], by="patient_id")

levels(all_bloods_table_long_BAs$active) = treatment.labs
```

Test in NHANES IV: Supplementary Table 3

```r
kdm2_nhanesiv <- kdm_nhanes_no_CA(biomarkers = blood_BMs_final_clinic_reduced)
agevar = c("kdm_advance0","kdm2_advance")
label = c("Original KDM\nBiological Age",
          "CCMR One Modified-KDM\nBiological Age")
table_surv(kdm2_nhanesiv$data, agevar, label)
table_health(kdm2_nhanesiv$data, agevar, outcome = c("health","adl","lnwalk","grip_scaled"), label)$table

#Same without lymphocyte %
kdm2_nhanesiv <- kdm_nhanes_no_CA(biomarkers = blood_BMs_final_clinic_reduced[blood_BMs_final_clinic_reduced != "lymph"])
agevar = c("kdm_advance0","kdm2_advance")
label = c("Original KDM\nBiological Age",
          "CCMR One Modified-KDM\nw/o lymph %")
table_surv(kdm2_nhanesiv$data, agevar, label)
table_health(kdm2_nhanesiv$data, agevar, outcome = c("health","adl","lnwalk","grip_scaled"), label)$table


#Make wide dataframe for bloods
id_cols_blood <- c("patient_id", "site", "gender", "active", "active.switched")

all_bloods_table_wide_BAs <- all_bloods_table_long_BAs[,c(id_cols_blood, "timepoint", "age", "kdm2", "kdm2_advance", "kdm2_no_lymph", "k
dm2_no_lymph_advance", "trig")] %>% reshape(direction = "wide",
                              timevar="timepoint",
                              idvar=id_cols_blood)
rownames(all_bloods_table_wide_BAs) <- NULL

all_bloods_table_wide_BAs$kdm2_advance.change.6 <- all_bloods_table_wide_BAs$kdm2_advance.6 - all_bloods_table_wide_BAs$kdm2_advance.0
all_bloods_table_wide_BAs$kdm2_advance.change.9 <- all_bloods_table_wide_BAs$kdm2_advance.9 - all_bloods_table_wide_BAs$kdm2_advance.0

all_bloods_table_wide_BAs$kdm2_no_lymph_advance.change.6 <- all_bloods_table_wide_BAs$kdm2_no_lymph.6 - all_bloods_table_wide_BAs$kdm2_n
o_lymph.0
```

```
# Add KDM to trial outcome datasets
# Relabel kdm2 as kdm for ease
bline_ITT <- bline_ITT %>% merge(all_bloods_table_wide_BAs[, c("patient_id", "kdm2.0", "kdm2_advance.0", "kdm2_advance.change.6", "kdm2_
no_lymph.0", "kdm2_no_lymph_advance.0", "kdm2_no_lymph_advance.change.6")] %>% dplyr::rename("kdm"="kdm2.0",

"kdm_advance"="kdm2_advance.0",
                                                                                                                     "kdm_advance.change"
="kdm2_advance.change.6",
                                                                                                                     "kdm_no_lymph"="kdm2
_no_lymph.0",

"kdm_no_lymph_advance"="kdm2_no_lymph_advance.0",
                                                                                                                     "kdm_no_lymph_advanc
e.change"="kdm2_no_lymph_advance.change.6"),
                    by="patient_id", all.x = TRUE)

bline_long_eyes_ITT <- bline_long_eyes_ITT %>% merge(all_bloods_table_wide_BAs[, c("patient_id", "kdm2.0", "kdm2_advance.0", "kdm2_advan
ce.change.6", "kdm2_no_lymph.0", "kdm2_no_lymph_advance.0", "kdm2_no_lymph_advance.change.6")] %>% dplyr::rename("kdm"="kdm2.0",
                                                                                                                     "kdm_advance"="kdm2_
advance.0",
                                                                                                                     "kdm_advance.change"
="kdm2_advance.change.6",
                                                                                                                     "kdm_no_lymph"="kdm2
_no_lymph.0",

"kdm_no_lymph_advance"="kdm2_no_lymph_advance.0",
                                                                                                                     "kdm_no_lymph_advanc
e.change"="kdm2_no_lymph_advance.change.6"),
                    by="patient_id", all.x = TRUE)

bline_ITT_bypatient <- bline_ITT_bypatient %>% merge(all_bloods_table_wide_BAs[, c("patient_id", "kdm2.0", "kdm2_advance.0", "kdm2_advan
ce.change.6", "kdm2_no_lymph.0", "kdm2_no_lymph_advance.0", "kdm2_no_lymph_advance.change.6")] %>% dplyr::rename("kdm"="kdm2.0",
                                                                                                                     "kdm_advance"="kdm2_
advance.0",
                                                                                                                     "kdm_advance.change"
="kdm2_advance.change.6",
                                                                                                                     "kdm_no_lymph"="kdm2
_no_lymph.0",

"kdm_no_lymph_advance"="kdm2_no_lymph_advance.0",
                                                                                                                     "kdm_no_lymph_advanc
e.change"="kdm2_no_lymph_advance.change.6"),
                    by="patient_id", all.x = TRUE)
```

3. Brain and blood age at baseline

Plot against CA: FIG 1A-B

```
#Baseline ages for paper (Figure 1A-B)

fig_brainage_baseline <- (ggplot()
    + theme_classic()
    + geom_point(data=brainage_all_long[brainage_all_long$timepoint==0,], aes(x = age, y = brainage), colour = "#FF9600", alpha = 0.7)
    + geom_abline(intercept = 0, slope = 1, color="#888888", linetype="dashed")
    + labs(x = "Chronological age", y = bquote(BA[MRI]))
    + coord_cartesian(ylim = c(0, 80), xlim=c(0, 80))
    )

fig_kdm2_baseline <- (ggplot()
    + theme_classic()
    + geom_point(data=all_bloods_table_long_BAs[all_bloods_table_long_BAs$timepoint==0,], aes(x = age, y = kdm2), colour = "#FF9600", al
pha = 0.7)
    + geom_abline(intercept = 0, slope = 1, color="#888888", linetype="dashed")
    + labs(x = "Chronological age", y = bquote(BA[Blood]))
    + coord_cartesian(ylim = c(0, 80), xlim=c(0, 80))
    )

fig_brainage_baseline

fig_kdm2_baseline

pdf(file="OutputForPaper/baseline_BAs.pdf", width=3, height=3)
fig_brainage_baseline
fig_kdm2_baseline
dev.off()

#Mean and SD at baseline - brain age
sprintf("Brain age advancement mean: %.01f years", mean(brainage_all$brainage_advance.0))
sprintf("Brain age advancement SD: %.01f years", sd(brainage_all$brainage_advance.0))

#Mean and SD at baseline - KDM age
sprintf("KDM age advancement mean: %.01f years", mean(all_bloods_table_long_BAs[all_bloods_table_long_BAs$timepoint==0,"kdm2_advance"],
na.rm=TRUE))
sprintf("KDM age advancement SD: %.01f years", sd(all_bloods_table_long_BAs[all_bloods_table_long_BAs$timepoint==0,"kdm2_advance"], na.r
m=TRUE))
```

Plot BA adv against each other: FIG 1C

```
model_kdm_brainage_adv <- lm(brainage_advance ~ kdm_advance, data = bline_ITT_bypatient)
```

```
fig_brainage_kdm_adv_byCA <- (ggplot()
  + theme_classic()
  + geom_point(data=bline_ITT_bypatient, aes(x = kdm_advance, y = brainage_advance, colour = age), alpha = 1)
  + scale_colour_gradientn(colours = c("purple", "orange"), name = "CA")
  + geom_abline(intercept = model_kdm_brainage_adv$coefficients["(Intercept)"], slope = model_kdm_brainage_adv$coefficients["kdm_advance
"], color="#888888", linetype="dashed")
  + labs(x = "Blood-based BA advancement (years)", y = "MRI brain age advancement (years)")
  )

pdf(file="OutputForPaper/fig_brainage_kdm_adv.pdf", width=3.5, height=3)
fig_brainage_kdm_adv_byCA
dev.off()

sprintf("KDMAge vs CA: r = %f", cor(bline_ITT_bypatient$kdm, bline_ITT_bypatient$Age.frac, use = "complete.obs"))
sprintf("BrainAge vs CA: r = %f", cor(bline_ITT_bypatient$brainage, bline_ITT_bypatient$Age.frac, use = "complete.obs"))
sprintf("KDMAge vs BrainAge: r = %f", cor(bline_ITT_bypatient$kdm, bline_ITT_bypatient$brainage, use = "complete.obs"))

print("---")
print("Advancement:")
sprintf("Overall: r = %f", cor(bline_ITT_bypatient$kdm_advance, bline_ITT_bypatient$brainage_advance, use = "complete.obs"))
cor.test(bline_ITT_bypatient$kdm_advance, bline_ITT_bypatient$brainage_advance)

fig_brainage_kdm_adv_byCA
```

EDSS and BA at baseline

```
#(Adjusting for CA)
coeff.model_edss_bybrainageadv_adj <- summary(lm(EDSS.score.at.baseline ~ brainage_advance + Age.frac, data = bline_ITT_bypatient))$coef
ficients["brainage_advance",c("Estimate", "Std. Error", "Pr(>|t|)")]

sprintf("EDSS; beta for raw brainage = %.03f (%.03f, %.03f); p = %.03f",
        coeff.model_edss_bybrainageadv_adj[1],
        (coeff.model_edss_bybrainageadv_adj[1]-1.96*coeff.model_edss_bybrainageadv_adj[2]),
        (coeff.model_edss_bybrainageadv_adj[1]+1.96*coeff.model_edss_bybrainageadv_adj[2]),
        coeff.model_edss_bybrainageadv_adj[3])


coeff.model_edss_bykdmadv_adj <- summary(lm(EDSS.score.at.baseline ~ kdm_advance + Age.frac, data = bline_ITT_bypatient))$coefficients["
kdm_advance",c("Estimate", "Std. Error", "Pr(>|t|)")]

sprintf("EDSS; beta for raw brainage = %.03f (%.03f, %.03f); p = %.03f",
        coeff.model_edss_bykdmadv_adj[1],
        (coeff.model_edss_bykdmadv_adj[1]-1.96*coeff.model_edss_bykdmadv_adj[2]),
        (coeff.model_edss_bykdmadv_adj[1]+1.96*coeff.model_edss_bykdmadv_adj[2]),
        coeff.model_edss_bykdmadv_adj[3])
```

4. Longitudinal assessment of brain age

Brainage trajectory by participant: FIG 2A

```
brainage_change_summary_data <- brainage_all_long[,c("timepoint", "active", "brainage_advance")] %>%
                 group_by(active, timepoint) %>%
                 dplyr::summarise(brainage_advance = mean(brainage_advance)) %>%
  merge(brainage_all_long[,c("timepoint", "active", "brainage_advance")] %>%
                 group_by(active, timepoint) %>%
                 dplyr::summarise(sem = (sd(brainage_advance)/sqrt(length(brainage_advance)))),
        by = c("timepoint", "active"))

brainage_change_summary_data$CI_low <- brainage_change_summary_data$brainage_advance - 1.96 * brainage_change_summary_data$sem
brainage_change_summary_data$CI_hi <- brainage_change_summary_data$brainage_advance + 1.96 * brainage_change_summary_data$sem

fig_brainage_change <- (ggplot()
  + theme_classic()
  + geom_hline(yintercept=0, colour="DarkGray")

  + geom_line(data=brainage_all_long, aes(x = timepoint, y = brainage_advance, group = patient_id, colour = active), alpha =0.3)
  + geom_point(data=brainage_all_long, aes(x = timepoint, y = brainage_advance, group = patient_id, colour = active), alpha =0.3)
  + geom_line(data=brainage_change_summary_data, aes(x = timepoint, y = brainage_advance, colour = active), alpha =1, size=1)
  + geom_point(data=brainage_change_summary_data, aes(x = timepoint, y = brainage_advance), colour="#5d5d5d", alpha =1, shape=18, size =
2, fill = "white")
  + geom_errorbar(data=brainage_change_summary_data, aes(x = timepoint, ymin=CI_low, ymax=CI_hi), colour="#5d5d5d", width=.2)

  + facet_wrap(~active)

  + labs(x = "Timepoint (months)", y = "MRI brain age advancement")
  + theme(legend.position = "none", plot.title = element_text(hjust = 0.5), plot.subtitle = element_text(hjust = 0.5))
  + theme(strip.background = element_blank())
)

pdf(file="OutputForPaper/fig_brainage_change.pdf", width=4, height=3)
fig_brainage_change
dev.off()

fig_brainage_change
```

Brainage change (+/- new lesions): FIG 2B

```
mri_mean_interval = mean(brainage_all$age.6 - brainage_all$age.0)
```

```r
#All ITT
brainage_change_summary_data_difference <- brainage_all[,c("active", "brainage.change")] %>%
                 group_by(active) %>%
                 dplyr::summarise(mean = mean(brainage.change)) %>%
  merge(brainage_all[,c("active", "brainage.change")] %>%
                 group_by(active) %>%
                 dplyr::summarise(sem = (sd(brainage.change)/sqrt(length(brainage.change)))),
        by = "active")  %>%
  merge(brainage_all[,c("active", "brainage.change")] %>%
                 group_by(active) %>%
                 dplyr::summarise(sd = (sd(brainage.change))),
        by = "active")

brainage_change_summary_data_difference$CI_low <- brainage_change_summary_data_difference$mean - 1.96 * brainage_change_summary_data_dif
ference$sem
brainage_change_summary_data_difference$CI_high <- brainage_change_summary_data_difference$mean + 1.96 * brainage_change_summary_data_di
fference$sem

#Only those with radiologically stable disease (ie. exclude n=5 with new lesions)
new_lesion_ids <- bline_ITT_bypatient[bline_ITT_bypatient$Number.of.patient.s.new..follow.up..lesions>0, "patient_id"]
brainage_all[brainage_all$patient_id %in% new_lesion_ids,]

brainage_change_summary_data_difference_exc <- brainage_all[!brainage_all$patient_id %in% new_lesion_ids,c("active", "brainage.change")]
%>%
                 group_by(active) %>%
                 dplyr::summarise(mean = mean(brainage.change)) %>%
  merge(brainage_all[!brainage_all$patient_id %in% new_lesion_ids,c("active", "brainage.change")] %>%
                 group_by(active) %>%
                 dplyr::summarise(sem = (sd(brainage.change)/sqrt(length(brainage.change)))),
        by = "active") %>%
  merge(brainage_all[!brainage_all$patient_id %in% new_lesion_ids,c("active", "brainage.change")] %>%
                 group_by(active) %>%
                 dplyr::summarise(sd = (sd(brainage.change))),
        by = "active")

brainage_change_summary_data_difference_exc$CI_low <- brainage_change_summary_data_difference_exc$mean - 1.96 * brainage_change_summary_
data_difference_exc$sem
brainage_change_summary_data_difference_exc$CI_high <- brainage_change_summary_data_difference_exc$mean + 1.96 * brainage_change_summary
_data_difference_exc$sem


fig_brainage_change_short <- (ggplot()
  + theme_classic()
  + geom_hline(yintercept=0, colour="DarkGray")
  + geom_hline(yintercept=mri_mean_interval, colour="DarkGray")

  + geom_point(data=brainage_all[!brainage_all$patient_id %in% new_lesion_ids,], aes(x = active, y = brainage.change, group = patient_id
, colour = active), alpha =0.6, position = position_jitter(width = 0.1, seed = 0))

  + geom_errorbar(data=brainage_change_summary_data_difference_exc, aes(x = active, ymin=CI_low, ymax=CI_high), colour="black", width=.0
8)
  + geom_point(data=brainage_change_summary_data_difference_exc, aes(x = active, y = mean), colour="black", alpha =1, shape=18, size = 2
, fill = "white")

  + labs(title = "", subtitle = "", x = "", y = "6-month change in MRI brain age")
  + theme(legend.position = "none", plot.title = element_text(hjust = 0.5), plot.subtitle = element_text(hjust = 0.5))
)

pdf(file="OutputForPaper/fig_brainage_change_short.pdf", width=3, height=3)
fig_brainage_change_short
dev.off()

fig_brainage_change_short

brainage_change_summary_data_difference
brainage_change_summary_data_difference_exc

#Linear model for brainage change bex vs placebo (adjusting for baseline value)
model_brainage_change <- lm(brainage_advance.change ~ active + brainage_advance.0,
     data = brainage_all)

diff <- summary(model_brainage_change)$coefficients["activeBexarotene","Estimate"]
SE <- summary(model_brainage_change)$coefficients["activeBexarotene","Std. Error"]
p <- summary(model_brainage_change)$coefficients["activeBexarotene","Pr(>|t|)"]
print(sprintf("Adjusted bexarotene brainage effect: %.02f years [CI: %.02f to %.02f], p = %.03f", diff, (diff-1.96*SE), (diff+1.96*SE),
p))

model_brainage_change_exc <- update(model_brainage_change, .~., data = brainage_all[!brainage_all$patient_id %in% new_lesion_ids,])

diff <- summary(model_brainage_change_exc)$coefficients["activeBexarotene","Estimate"]
SE <- summary(model_brainage_change_exc)$coefficients["activeBexarotene","Std. Error"]
p <- summary(model_brainage_change_exc)$coefficients["activeBexarotene","Pr(>|t|)"]
print(sprintf("Adjusted bexarotene brainage effect (brains with new lesions excluded): %.02f years [CI: %.02f to %.02f], p = %.03f", dif
f, (diff-1.96*SE), (diff+1.96*SE), p))

print("=====")

# Paired t-test for before vs after treatment:
# All ITT bexarotene
t.test(brainage_all[brainage_all$active=="Bexarotene","brainage_advance.6"],
       brainage_all[brainage_all$active=="Bexarotene","brainage_advance.0"],
       paired = TRUE, alternative = "two.sided")

# No new lesions bexarotene
t.test(brainage_all[brainage_all$active=="Bexarotene" & !brainage_all$patient_id %in% new_lesion_ids,"brainage_advance.6"],
       brainage_all[brainage_all$active=="Bexarotene" & !brainage_all$patient_id %in% new_lesion_ids,"brainage_advance.0"],
       paired = TRUE, alternative = "two.sided")
```

```
# No new lesions placebo
t.test(brainage_all[brainage_all$active=="Placebo" & !brainage_all$patient_id %in% new_lesion_ids,"brainage_advance.6"],
       brainage_all[brainage_all$active=="Placebo" & !brainage_all$patient_id %in% new_lesion_ids,"brainage_advance.0"],
       paired = TRUE, alternative = "two.sided")

print("=====")

#t-test for comparison of brainage change
#Bexarotene
t.test(brainage_all[!brainage_all$patient_id %in% new_lesion_ids & brainage_all$active=="Bexarotene","brainage.change"], mu = 0.5, alter
native = "two.sided")

#Placebo
t.test(brainage_all[!brainage_all$patient_id %in% new_lesion_ids & brainage_all$active=="Placebo","brainage.change"], mu = 0.5, alternat
ive = "two.sided")


#Review those with >1SD reduction in brainage
sd_bex_brainage_advance.change <- sd(bline_ITT_bypatient[bline_ITT_bypatient$Treatment==" Bexarotene","brainage_advance.change"])

best_brainage_adv <- bline_ITT_bypatient[bline_ITT_bypatient$Treatment==" Bexarotene" & bline_ITT_bypatient$brainage_advance.change < -s
d_bex_brainage_advance.change,"patient_id"]
not_best_brainage_adv <- bline_ITT_bypatient[bline_ITT_bypatient$Treatment==" Bexarotene" & bline_ITT_bypatient$brainage_advance.change
> -sd_bex_brainage_advance.change,"patient_id"]

bline_ITT_bypatient[bline_ITT_bypatient$patient_id %in% best_brainage_adv, c("age", "brainage_advance", "Gender", "site", "EDSS.score.at
.screening", "num_rel_2yr_prior")]

#X-squared for proportion female
chisq.test(rbind(c(sum(bline_ITT_bypatient[bline_ITT_bypatient$patient_id %in% best_brainage_adv, c("sex")] == 1),
                   sum(bline_ITT_bypatient[bline_ITT_bypatient$patient_id %in% best_brainage_adv, c("sex")] == 0)),
                   c(sum(bline_ITT_bypatient[bline_ITT_bypatient$patient_id %in% not_best_brainage_adv, c("sex")] == 1),
                   sum(bline_ITT_bypatient[bline_ITT_bypatient$patient_id %in% not_best_brainage_adv, c("sex")] == 0))))
```

Compare change in brain age with remyelination in different regions: FIG 2C

```
les_location.labs <- c("Periventricular", "Deep WM", "Juxtacortical", "Leucocortical", "Cortical GM", "Deep GM", "Mixed DGM + WM", "Brai
nstem", "Cerebellum")
names(les_location.labs) <- c(0,1,2,3,4,5,6,7,8)


#Mixed linear model
brainage_mtr_correlation <- data.frame("Location"=c("All", les_location.labs), order = c(11,9,8,7,6,5,4,3,2,1), beta = NA, SE = NA, CI.l
ow = NA, CI.high = NA, p = NA)

brainage_mtr_change_overall_model <- lmer(ch_wles_mtr ~ brainage_advance.change + bl_wholeles_mtr_std + active + (1|id), data = bline_IT
T)#[bline_ITT$active==1,])

brainage_mtr_correlation[brainage_mtr_correlation$Location=="All", c("beta", "SE", "p")] <- summary(brainage_mtr_change_overall_model)$c
oefficients["brainage_advance.change", c("Estimate", "Std. Error", "Pr(>|t|)")]

# Loop through each region

fun_1 <- "lmer(ch_wles_mtr ~ brainage_advance.change * "

fun_2 <- " + bl_wholeles_mtr_std + brainage + active.switched
                              + (1|id)
                              ,data = bline_ITT)"   #[bline_ITT$active==1,])"

for (loc in 1:length (les_location.labs)){
  loc_string = les_location.labs[[loc]]
  switched <- paste("les_location.switched0", (loc-1), sep="")
  if(loc==1){
    switched <- "les_location"
    }

  loc_model <- eval(parse(text = paste(fun_1, switched, fun_2, sep = "")))

  brainage_mtr_correlation[brainage_mtr_correlation$Location==loc_string, c("beta", "SE", "p")] <- summary(loc_model)$coefficients["brai
nage_advance.change", c("Estimate", "Std. Error", "Pr(>|t|)")]

}

brainage_mtr_correlation$CI.low <- brainage_mtr_correlation$beta - 1.96 * brainage_mtr_correlation$SE
brainage_mtr_correlation$CI.high <- brainage_mtr_correlation$beta + 1.96 * brainage_mtr_correlation$SE

brainage_mtr_correlation[,-c(1,2)] = round(brainage_mtr_correlation[,-c(1,2)],3)
brainage_mtr_correlation$Location = factor(brainage_mtr_correlation$Location, levels = rev(les_location.labs))
brainage_mtr_correlation

fig_brainage_change_regionalmtr <- (ggplot(data = brainage_mtr_correlation[-1,], aes(y=Location, x=beta, xmin=CI.low, xmax=CI.high, colo
ur=p<0.05))
  + theme_classic()
  + geom_vline(xintercept=0, colour="DarkGray")
  + geom_point()
  + geom_errorbarh(height=.1)
  + scale_color_manual(values=c("#999999", "#FF9600"))
  + ylab("")
  + xlab(expression(beta~"for MRI brain age advancement (pu/year)"))
  + theme(legend.position = "none", plot.title = element_text(hjust = 0.5), plot.subtitle = element_text(hjust = 0.5))
  )

fig_brainage_change_regionalmtr


pdf(file="OutputForPaper/fig_brainage_change_regionalmtr.pdf", width=4.5, height=3)
fig_brainage_change_regionalmtr
dev.off()
```

5. Longitidunal assessment of blood age

Blood BA trajectory by participant: FIG S3

```
kdm2_change_summary_data <- all_bloods_table_long_BAs[all_bloods_table_long_BAs$site=="Cambridge" & !is.na(all_bloods_table_long_BAs$kdm2_advance),
                                                       c("timepoint", "active", "kdm2_advance")] %>%
                 group_by(active, timepoint) %>%
                 dplyr::summarise(kdm2_advance = mean(kdm2_advance)) %>%
  merge(all_bloods_table_long_BAs[all_bloods_table_long_BAs$site=="Cambridge" & !is.na(all_bloods_table_long_BAs$kdm2_advance),c("timepoint", "active", "kdm2_advance")] %>%
                 group_by(active, timepoint) %>%
                 dplyr::summarise(sem = (sd(kdm2_advance)/sqrt(length(kdm2_advance)))),
        by = c("timepoint", "active"))

kdm2_change_summary_data$CI_low <- kdm2_change_summary_data$kdm2_advance - 1.96 * kdm2_change_summary_data$sem
kdm2_change_summary_data$CI_hi <- kdm2_change_summary_data$kdm2_advance + 1.96 * kdm2_change_summary_data$sem


fig_kdm2_change <- (ggplot()
  + theme_classic()
  + geom_hline(yintercept=0, colour="DarkGray")

  + geom_line(data=all_bloods_table_long_BAs[all_bloods_table_long_BAs$site=="Cambridge",], aes(x = timepoint, y = kdm2_advance, group = patient_id, colour = active), alpha =0.3)
  + geom_point(data=all_bloods_table_long_BAs[all_bloods_table_long_BAs$site=="Cambridge",], aes(x = timepoint, y = kdm2_advance, group = patient_id, colour = active), alpha =0.3)
  + geom_line(data=kdm2_change_summary_data, aes(x = timepoint, y = kdm2_advance, colour = active), alpha =1, size=1)
  + geom_point(data=kdm2_change_summary_data, aes(x = timepoint, y = kdm2_advance), colour="#5d5d5d", alpha =1, shape=18, size = 2, fill = "white")
  + geom_errorbar(data=kdm2_change_summary_data, aes(x = timepoint, ymin=CI_low, ymax=CI_hi), colour="#5d5d5d", width=.2)

  + facet_wrap(~active)

  + labs(x = "Timepoint (months)", y = "Blood-based biological\nage advancement")
  + theme(legend.position = "none", plot.title = element_text(hjust = 0.5), plot.subtitle = element_text(hjust = 0.5))
  + theme(strip.background = element_blank())
  + coord_cartesian(ylim = c(-25, 70))
  + scale_x_continuous(breaks=seq(0,9,3))
)

fig_kdm2_change


pdf(file="OutputForPaper/fig_kdm2_change.pdf", width=4, height=3)
fig_kdm2_change
dev.off()


model_kdm_change_6m <- lm(kdm2_advance.change.6 ~ active + kdm2.0,
     data = all_bloods_table_wide_BAs[all_bloods_table_wide_BAs$site=="Cambridge",])

diff <- summary(model_kdm_change_6m)$coefficients["activeBexarotene","Estimate"]
SE <- summary(model_kdm_change_6m)$coefficients["activeBexarotene","Std. Error"]
p <- summary(model_kdm_change_6m)$coefficients["activeBexarotene","Pr(>|t|)"]
print(sprintf("Adjusted bexarotene KDM effect (6 months): %.02f years [CI: %.02f to %.02f], p = %.03f", diff, (diff-1.96*SE), (diff+1.96*SE), p))

model_kdm_change_6m_trig <- lm(kdm2_advance.change.6 ~ active + kdm2.0 + trig.6,
     data = all_bloods_table_wide_BAs[all_bloods_table_wide_BAs$site=="Cambridge",])

diff <- summary(model_kdm_change_6m_trig)$coefficients["activeBexarotene","Estimate"]
SE <- summary(model_kdm_change_6m_trig)$coefficients["activeBexarotene","Std. Error"]
p <- summary(model_kdm_change_6m_trig)$coefficients["activeBexarotene","Pr(>|t|)"]
print(sprintf("Adjusted bexarotene KDM effect (6 months) adjusted for triglycerides: %.02f years [CI: %.02f to %.02f], p = %.03f", diff, (diff-1.96*SE), (diff+1.96*SE), p))

model_kdm_change_9m <- lm(kdm2_advance.change.9 ~ active + kdm2.0,
     data = all_bloods_table_wide_BAs[all_bloods_table_wide_BAs$site=="Cambridge",])

diff <- summary(model_kdm_change_9m)$coefficients["activeBexarotene","Estimate"]
SE <- summary(model_kdm_change_9m)$coefficients["activeBexarotene","Std. Error"]
p <- summary(model_kdm_change_9m)$coefficients["activeBexarotene","Pr(>|t|)"]
print(sprintf("Adjusted bexarotene KDM effect (9 months): %.02f years [CI: %.02f to %.02f], p = %.03f", diff, (diff-1.96*SE), (diff+1.96*SE), p))

print(sprintf("Population for longitudinal KDM effect: n = %.f", sum(1*!is.na(all_bloods_table_wide_BAs[,"kdm2_advance.change.6"]))))
```

Individual blood biomarker changes: Supplementary Table S4

```
treatment_effects_blood <- data.frame(biomarker=blood_BMs_final_clinic_reduced, estimate=NA, se=NA, t=NA, p=NA)

for(biomarker in blood_BMs_final_clinic_reduced){
  summary_data = all_bloods_table_long_BAs[all_bloods_table_long_BAs$site=="Cambridge" & all_bloods_table_long_BAs$timepoint==0, c("patient_id", biomarker, "active.switched")] %>%
  merge(all_bloods_table_long_BAs[all_bloods_table_long_BAs$site=="Cambridge" & all_bloods_table_long_BAs$timepoint==6, c("patient_id", biomarker)], by = "patient_id") %>%
  dplyr::rename("m6"=paste0(biomarker, ".y"), "m0"=paste0(biomarker, ".x"))

  treatment_effects_blood[treatment_effects_blood$biomarker == biomarker, 2:5] <-
    summary(glm(m6 ~ m0 + active.switched, data = summary_data))$coefficients["active.switched1",]
}

treatment_effects_blood$estimate = -treatment_effects_blood$estimate #(as using treatment.switched here)
treatment_effects_blood
```

6. VEP Outcomes

```
# Create dataframe to exclude those with recent ON: bline_long_eyes_ITT_no5yON

bline_long_eyes_ITT_no5yON <- bline_long_eyes_ITT

for(i in 1:nrow(bline_long_eyes_ITT_no5yON)) {
  if (bline_long_eyes_ITT_no5yON$eye[i]=="ch_veplat100_L") {
    if (!is.na(bline_long_eyes_ITT_no5yON$TimesinceONLeye[i])) {
      if (bline_long_eyes_ITT_no5yON$TimesinceONLeye[i]<=1825) {bline_long_eyes_ITT_no5yON$ch_veplat100[i] <- NA}}}
  if (bline_long_eyes_ITT_no5yON$eye[i]=="ch_veplat100_R") {
    if (!is.na(bline_long_eyes_ITT_no5yON$TimesinceONReye[i])) {
      if (bline_long_eyes_ITT_no5yON$TimesinceONReye[i]<=1825) {bline_long_eyes_ITT_no5yON$ch_veplat100[i] <- NA}}}
  }

#Exclude individual eye with ON during the trial
bline_long_eyes_ITT_no5yON[bline_long_eyes_ITT_no5yON$uniqueID3=="X112055_Y1344421","ch_veplat100"] <- NA
```

Check numbers - For Supplementary Table 1

```
print(sprintf("All eyes in study: %.0f eyes, (%.0f people)",
              length(bline_long_eyes_ITT[, "patient_id"]),
              length(unique(bline_long_eyes_ITT[, "patient_id"]))))
print(sprintf("All eyes in study (bexarotene): %.0f eyes, (%.0f people)",
              length(bline_long_eyes_ITT[bline_long_eyes_ITT$Treatment==" Bexarotene", "patient_id"]),
              length(unique(bline_long_eyes_ITT[bline_long_eyes_ITT$Treatment==" Bexarotene", "patient_id"]))))
print(sprintf("All eyes in study (placebo): %.0f eyes, (%.0f people)",
              length(bline_long_eyes_ITT[bline_long_eyes_ITT$Treatment==" Placebo", "patient_id"]),
              length(unique(bline_long_eyes_ITT[bline_long_eyes_ITT$Treatment==" Placebo", "patient_id"]))))

print("")
print(sprintf("All eyes for analysis: %.0f eyes, (%.0f people)",
              length(bline_long_eyes_ITT[!is.na(bline_long_eyes_ITT$ch_veplat100), "patient_id"]),
              length(unique(bline_long_eyes_ITT[!is.na(bline_long_eyes_ITT$ch_veplat100), "patient_id"]))))
print(sprintf("All eyes for analysis (bexarotene): %.0f eyes, (%.0f people)",
              length(bline_long_eyes_ITT[!is.na(bline_long_eyes_ITT$ch_veplat100) & bline_long_eyes_ITT$Treatment==" Bexarotene", "patie
nt_id"]),
              length(unique(bline_long_eyes_ITT[!is.na(bline_long_eyes_ITT$ch_veplat100) & bline_long_eyes_ITT$Treatment==" Bexarotene",
"patient_id"]))))
print(sprintf("All eyes for analysis (placebo): %.0f eyes, (%.0f people)",
              length(bline_long_eyes_ITT[!is.na(bline_long_eyes_ITT$ch_veplat100) & bline_long_eyes_ITT$Treatment==" Placebo", "patient_
id"]),
              length(unique(bline_long_eyes_ITT[!is.na(bline_long_eyes_ITT$ch_veplat100) & bline_long_eyes_ITT$Treatment==" Placebo", "p
atient_id"]))))

print("")
print(sprintf("All eyes for analysis >118ms: %.0f eyes, (%.0f people)",
              length(bline_long_eyes_ITT[!is.na(bline_long_eyes_ITT$ch_veplat100) & bline_long_eyes_ITT$bin_bline_P100==1, "patient_id"]
),
              length(unique(bline_long_eyes_ITT[!is.na(bline_long_eyes_ITT$ch_veplat100) & bline_long_eyes_ITT$bin_bline_P100==1, "patie
nt_id"]))))
print(sprintf("All eyes for analysis >118ms (bexarotene): %.0f eyes, (%.0f people)",
              length(bline_long_eyes_ITT[!is.na(bline_long_eyes_ITT$ch_veplat100) & bline_long_eyes_ITT$bin_bline_P100==1 & bline_long_e
yes_ITT$Treatment==" Bexarotene", "patient_id"]),
              length(unique(bline_long_eyes_ITT[!is.na(bline_long_eyes_ITT$ch_veplat100) & bline_long_eyes_ITT$bin_bline_P100==1 & bline
_long_eyes_ITT$Treatment==" Bexarotene", "patient_id"]))))
print(sprintf("All eyes for analysis >118ms (placebo): %.0f eyes, (%.0f people)",
              length(bline_long_eyes_ITT[!is.na(bline_long_eyes_ITT$ch_veplat100) & bline_long_eyes_ITT$bin_bline_P100==1 & bline_long_e
yes_ITT$Treatment==" Placebo", "patient_id"]),
              length(unique(bline_long_eyes_ITT[!is.na(bline_long_eyes_ITT$ch_veplat100) & bline_long_eyes_ITT$bin_bline_P100==1 & bline
_long_eyes_ITT$Treatment==" Placebo", "patient_id"]))))


print("")
print(sprintf("No recent ON eyes for analysis >118ms: %.0f eyes, (%.0f people)",
              length(bline_long_eyes_ITT_no5yON[!is.na(bline_long_eyes_ITT_no5yON$ch_veplat100) & bline_long_eyes_ITT_no5yON$bin_bline_P
100==1, "patient_id"]),
              length(unique(bline_long_eyes_ITT_no5yON[!is.na(bline_long_eyes_ITT_no5yON$ch_veplat100) & bline_long_eyes_ITT_no5yON$bin_
bline_P100==1, "patient_id"]))))
print(sprintf("No recent ON eyes for analysis >118ms (bexarotene): %.0f eyes, (%.0f people)",
              length(bline_long_eyes_ITT_no5yON[!is.na(bline_long_eyes_ITT_no5yON$ch_veplat100) & bline_long_eyes_ITT_no5yON$bin_bline_P
100==1 & bline_long_eyes_ITT_no5yON$Treatment==" Bexarotene", "patient_id"]),
              length(unique(bline_long_eyes_ITT_no5yON[!is.na(bline_long_eyes_ITT_no5yON$ch_veplat100) & bline_long_eyes_ITT_no5yON$bin_
bline_P100==1 & bline_long_eyes_ITT_no5yON$Treatment==" Bexarotene", "patient_id"]))))
print(sprintf("No recent ON eyes for analysis >118ms (placebo): %.0f eyes, (%.0f people)",
              length(bline_long_eyes_ITT_no5yON[!is.na(bline_long_eyes_ITT_no5yON$ch_veplat100) & bline_long_eyes_ITT_no5yON$bin_bline_P
100==1 & bline_long_eyes_ITT_no5yON$Treatment==" Placebo", "patient_id"]),
              length(unique(bline_long_eyes_ITT_no5yON[!is.na(bline_long_eyes_ITT_no5yON$ch_veplat100) & bline_long_eyes_ITT_no5yON$bin_
bline_P100==1 & bline_long_eyes_ITT_no5yON$Treatment==" Placebo", "patient_id"]))))
#NB 1 fewer eye in bexarotene & no recent ON & baseline >118 compared to table in trial manuscript (25 vs 26), as here we have also excl
uded the eye with ON during the trial

print("")
print(sprintf("Participants with no new lesions: %.0f", length(brainage_all[!brainage_all$patient_id %in% new_lesion_ids, "patient_id"])
))
print(sprintf("Participants with no new lesions (bexarotene): %.0f", length(brainage_all[!brainage_all$patient_id %in% new_lesion_ids &
brainage_all$active=="Bexarotene", "patient_id"])))
print(sprintf("Participants with no new lesions (placebo): %.0f", length(brainage_all[!brainage_all$patient_id %in% new_lesion_ids & bra
inage_all$active=="Placebo", "patient_id"])))
```

VEP and CA model; then modified for unadjusted and CA-adjusted BAs

```
modelvep_bex_ca <- lmer(ch_veplat100 ~ active.switched:bin_bline_P100.switched01:Age.frac
                              + active.switched:bin_bline_P100.switched01 #>118ms group = baseline
                              + active.switched:Age.frac
                              + bin_bline_P100.switched01:Age.frac
                              + active.switched
                              + bin_bline_P100.switched01
                              + bin_edss
                              + gender
                              + site
                              + Age.frac
                              + (1 | id),
                              data = bline_long_eyes_ITT)

modelvep_bex_ca_exc <- update(modelvep_bex_ca, ch_veplat100_no5yON ~ .)


#Brain age (unadjusted)
modelvep_bex_brainage_raw <- update(modelvep_bex_ca, . ~ . - Age.frac - active.switched:Age.frac - bin_bline_P100.switched01:Age.frac
                              - active.switched:bin_bline_P100.switched01:Age.frac
                              + brainage + active.switched:brainage + bin_bline_P100.switched01:brainage
                              + active.switched:bin_bline_P100.switched01:brainage)

modelvep_bex_brainage_raw_exc <- update(modelvep_bex_brainage_raw, . ~ ., data = bline_long_eyes_ITT_no5yON)


#Blood age (unadjusted)
modelvep_bex_kdm_raw <- update(modelvep_bex_ca, . ~ . - Age.frac - active.switched:Age.frac - bin_bline_P100.switched01:Age.frac
                              - active.switched:bin_bline_P100.switched01:Age.frac
                              + kdm + active.switched:kdm + bin_bline_P100.switched01:kdm
                              + active.switched:bin_bline_P100.switched01:kdm)

modelvep_bex_kdm_raw_exc <- update(modelvep_bex_kdm_raw, . ~ ., data = bline_long_eyes_ITT_no5yON)


#Brain age ADVANCEMENT (adjusted)
modelvep_bex_brainage_adj <- update(modelvep_bex_ca, . ~ . + brainage_advance + active.switched:brainage_advance + bin_bline_P100.switch
ed01:brainage_advance
                              + active.switched:bin_bline_P100.switched01:brainage_advance)

modelvep_bex_brainage_adj_exc <- update(modelvep_bex_brainage_adj, . ~ ., data = bline_long_eyes_ITT_no5yON)


#Blood age ADVANCEMENT (adjusted)
modelvep_bex_kdm_adj <- update(modelvep_bex_ca, . ~ . + kdm_advance + active.switched:kdm_advance + bin_bline_P100.switched01:kdm_advanc
e
                              + active.switched:bin_bline_P100.switched01:kdm_advance)

modelvep_bex_kdm_adj_exc <- update(modelvep_bex_kdm_adj, . ~ ., data = bline_long_eyes_ITT_no5yON)
```

Tabulate VEP outcome data: Supplementary Table 5

```
#CA
forest_vep_ca <- data.frame(criteria="", label = "", beta="", CI.low="", CI.high="", p="")

forest_vep_ca <- rbind(forest_vep_ca, c("ca_all", "All eyes",
  -summary(modelvep_bex_ca)$coefficients["Age.frac", "Estimate"],
  -(summary(modelvep_bex_ca)$coefficients["Age.frac", "Estimate"] + (1.96 * summary(modelvep_bex_ca)$coefficients["Age.frac", "Std. Erro
r"])),
  -(summary(modelvep_bex_ca)$coefficients["Age.frac", "Estimate"] - (1.96 * summary(modelvep_bex_ca)$coefficients["Age.frac", "Std. Erro
r"])),
  summary(modelvep_bex_ca)$coefficients["Age.frac", "Pr(>|t|)"]))

forest_vep_ca <- rbind(forest_vep_ca, c("ca_exc", "ON excluded",
  -summary(modelvep_bex_ca_exc)$coefficients["Age.frac", "Estimate"],
  -(summary(modelvep_bex_ca_exc)$coefficients["Age.frac", "Estimate"] + (1.96 * summary(modelvep_bex_ca_exc)$coefficients["Age.frac", "S
td. Error"])),
  -(summary(modelvep_bex_ca_exc)$coefficients["Age.frac", "Estimate"] - (1.96 * summary(modelvep_bex_ca_exc)$coefficients["Age.frac", "S
td. Error"])),
  summary(modelvep_bex_ca_exc)$coefficients["Age.frac", "Pr(>|t|)"]))

forest_vep_ca <- forest_vep_ca[-1,] %>% mutate_at(c("beta", "CI.low", "CI.high", "p"), as.numeric)
rownames(forest_vep_ca) <- NULL


#MRI brain age
forest_vep_brain <- data.frame(criteria="", label = "", beta="", CI.low="", CI.high="", p="")

forest_vep_brain <- rbind(forest_vep_brain, c("brain_all_raw", "All eyes",
  -summary(modelvep_bex_brainage_raw)$coefficients["brainage", "Estimate"],
  -(summary(modelvep_bex_brainage_raw)$coefficients["brainage", "Estimate"] + (1.96 * summary(modelvep_bex_brainage_raw)$coefficients["b
rainage", "Std. Error"])),
  -(summary(modelvep_bex_brainage_raw)$coefficients["brainage", "Estimate"] - (1.96 * summary(modelvep_bex_brainage_raw)$coefficients["b
rainage", "Std. Error"])),
  summary(modelvep_bex_brainage_raw)$coefficients["brainage", "Pr(>|t|)"]))

forest_vep_brain <- rbind(forest_vep_brain, c("brain_exc_raw", "ON excluded",
  -summary(modelvep_bex_brainage_raw_exc)$coefficients["brainage", "Estimate"],
  -(summary(modelvep_bex_brainage_raw_exc)$coefficients["brainage", "Estimate"] + (1.96 * summary(modelvep_bex_brainage_raw_exc)$coeffic
ients["brainage", "Std. Error"])),
  -(summary(modelvep_bex_brainage_raw_exc)$coefficients["brainage", "Estimate"] - (1.96 * summary(modelvep_bex_brainage_raw_exc)$coeffic
ients["brainage", "Std. Error"])),
  summary(modelvep_bex_brainage_raw_exc)$coefficients["brainage", "Pr(>|t|)"]))

forest_vep_brain <- rbind(forest_vep_brain, c("brain_all_adj", "All eyes (adjusted)",
```

```
  -summary(modelvep_bex_brainage_adj)$coefficients["brainage_advance", "Estimate"],
  -(summary(modelvep_bex_brainage_adj)$coefficients["brainage_advance", "Estimate"] + (1.96 * summary(modelvep_bex_brainage_adj)$coeffic
ients["brainage_advance", "Std. Error"])),
  -(summary(modelvep_bex_brainage_adj)$coefficients["brainage_advance", "Estimate"] - (1.96 * summary(modelvep_bex_brainage_adj)$coeffic
ients["brainage_advance", "Std. Error"])),
  summary(modelvep_bex_brainage_adj)$coefficients["brainage_advance", "Pr(>|t|)"]))

forest_vep_brain <- rbind(forest_vep_brain, c("brain_exc_adj", "ON excluded (adjusted)",
  -summary(modelvep_bex_brainage_adj_exc)$coefficients["brainage_advance", "Estimate"],
  -(summary(modelvep_bex_brainage_adj_exc)$coefficients["brainage_advance", "Estimate"] + (1.96 * summary(modelvep_bex_brainage_adj_exc)
$coefficients["brainage_advance", "Std. Error"])),
  -(summary(modelvep_bex_brainage_adj_exc)$coefficients["brainage_advance", "Estimate"] - (1.96 * summary(modelvep_bex_brainage_adj_exc)
$coefficients["brainage_advance", "Std. Error"])),
  summary(modelvep_bex_brainage_adj_exc)$coefficients["brainage_advance", "Pr(>|t|)"]))

forest_vep_brain <- forest_vep_brain[-1,] %>% mutate_at(c("beta", "CI.low", "CI.high", "p"), as.numeric)
rownames(forest_vep_brain) <- NULL


#Blood-based BA
forest_vep_kdm <- data.frame(criteria="", label = "", beta="", CI.low="", CI.high="", p="")

forest_vep_kdm <- rbind(forest_vep_kdm, c("kdm_all_raw", "All eyes",
  -summary(modelvep_bex_kdm_raw)$coefficients["kdm", "Estimate"],
  -(summary(modelvep_bex_kdm_raw)$coefficients["kdm", "Estimate"] + (1.96 * summary(modelvep_bex_kdm_raw)$coefficients["kdm", "Std. Erro
r"])),
  -(summary(modelvep_bex_kdm_raw)$coefficients["kdm", "Estimate"] - (1.96 * summary(modelvep_bex_kdm_raw)$coefficients["kdm", "Std. Erro
r"])),
  summary(modelvep_bex_kdm_raw)$coefficients["kdm", "Pr(>|t|)"]))

forest_vep_kdm <- rbind(forest_vep_kdm, c("kdm_exc_raw", "ON excluded",
  -summary(modelvep_bex_kdm_raw_exc)$coefficients["kdm", "Estimate"],
  -(summary(modelvep_bex_kdm_raw_exc)$coefficients["kdm", "Estimate"] + (1.96 * summary(modelvep_bex_kdm_raw_exc)$coefficients["kdm", "S
td. Error"])),
  -(summary(modelvep_bex_kdm_raw_exc)$coefficients["kdm", "Estimate"] - (1.96 * summary(modelvep_bex_kdm_raw_exc)$coefficients["kdm", "S
td. Error"])),
  summary(modelvep_bex_kdm_raw_exc)$coefficients["kdm", "Pr(>|t|)"]))

forest_vep_kdm <- rbind(forest_vep_kdm, c("kdm_all_adj", "All eyes (adjusted)",
  -summary(modelvep_bex_kdm_adj)$coefficients["kdm_advance", "Estimate"],
  -(summary(modelvep_bex_kdm_adj)$coefficients["kdm_advance", "Estimate"] + (1.96 * summary(modelvep_bex_kdm_adj)$coefficients["kdm_adva
nce", "Std. Error"])),
  -(summary(modelvep_bex_kdm_adj)$coefficients["kdm_advance", "Estimate"] - (1.96 * summary(modelvep_bex_kdm_adj)$coefficients["kdm_adva
nce", "Std. Error"])),
  summary(modelvep_bex_kdm_adj)$coefficients["kdm_advance", "Pr(>|t|)"]))

forest_vep_kdm <- rbind(forest_vep_kdm, c("kdm_exc_adj", "ON excluded (adjusted)",
  -summary(modelvep_bex_kdm_adj_exc)$coefficients["kdm_advance", "Estimate"],
  -(summary(modelvep_bex_kdm_adj_exc)$coefficients["kdm_advance", "Estimate"] + (1.96 * summary(modelvep_bex_kdm_adj_exc)$coefficients["
kdm_advance", "Std. Error"])),
  -(summary(modelvep_bex_kdm_adj_exc)$coefficients["kdm_advance", "Estimate"] - (1.96 * summary(modelvep_bex_kdm_adj_exc)$coefficients["
kdm_advance", "Std. Error"])),
  summary(modelvep_bex_kdm_adj_exc)$coefficients["kdm_advance", "Pr(>|t|)"]))

forest_vep_kdm <- forest_vep_kdm[-1,] %>% mutate_at(c("beta", "CI.low", "CI.high", "p"), as.numeric)
rownames(forest_vep_kdm) <- NULL

forest_vep <- forest_vep_ca %>%
  rbind(data.frame(criteria="blank1", label = "", beta=NA, CI.low=NA, CI.high=NA, p=NA)) %>%
  rbind(forest_vep_brain) %>%
  rbind(data.frame(criteria="blank2", label = "", beta=NA, CI.low=NA, CI.high=NA, p=NA)) %>%
  rbind(forest_vep_kdm)

forest_vep$criteria <- factor(forest_vep$criteria, levels = rev(forest_vep$criteria))

forest_vep
```

VEP plot: FIG 3

```
forest_vep_all <- forest_vep[forest_vep$criteria %in% c("ca_all", "blank1", "brain_all_raw", "brain_all_adj", "blank2", "kdm_all_raw", "
kdm_all_adj"),]

forest_vep_all$label <- c(" ", "", "Unadj.", "Adj.", "", "Unadj.", "Adj.")
forest_vep_all$shape <- c(1, "", 1, 2, "", 1, 2)

forest_vep_subfig_all <- (ggplot(data = forest_vep_all, aes(y=criteria, x=beta, xmin=CI.low, xmax=CI.high, colour=p<0.05))
  + theme_classic()
  + geom_vline(xintercept=0, colour="DarkGray")
  + geom_hline(yintercept="blank1", colour="#999999", linetype = "dashed")
  + geom_hline(yintercept="blank2", colour="#999999", linetype = "dashed")

  + geom_point(aes(shape=forest_vep_all$shape))
  + scale_shape_manual(values=c(0,16,17, 0))
  + geom_errorbarh(height=.1)

  + scale_y_discrete(label=rev(forest_vep_all$label))
  + scale_color_manual(values=c("#999999", "#FF9600"))

 # + scale_y_continuous(breaks = -4:9, label=forest_vep$label)

  + coord_cartesian(xlim=c(-1.1, 0.5))
  + scale_x_continuous(breaks=seq(-1, 1, 0.5))

  #+ scale_color_manual(values=c("#999999", "#00BFC4", "#F8766D", "#00BFC4", "#F8766D"))

  + labs(x = expression(beta~"for P100 latency reduction (ms / year)"), y = "",
        title = "",
```

```
       subtitle = "")
  + theme(legend.position = "none", plot.title = element_blank(), plot.subtitle = element_blank())
  )

forest_vep_subfig_all


forest_vep_noON <- forest_vep[forest_vep$criteria %in% c("ca_exc", "blank1", "brain_exc_raw", "brain_exc_adj", "blank2", "kdm_exc_raw",
"kdm_exc_adj"),]

forest_vep_noON$label <- c(" ", "", "Unadj.", "Adj.", "", "Unadj.", "Adj.")
forest_vep_noON$shape <- c(1, "", 1, 2, "", 1, 2)

forest_vep_subfig_noON <- (ggplot(data = forest_vep_noON, aes(y=criteria, x=beta, xmin=CI.low, xmax=CI.high, colour=p<0.05))
  + theme_classic()
  + geom_vline(xintercept=0, colour="DarkGray")
  + geom_hline(yintercept="blank1", colour="#999999", linetype = "dashed")
  + geom_hline(yintercept="blank2", colour="#999999", linetype = "dashed")

  + geom_point(aes(shape=forest_vep_noON$shape))
  + scale_shape_manual(values=c(0,16,17, 0))
  + geom_errorbarh(height=.1)

  + scale_y_discrete(label=rev(forest_vep_noON$label))
  + scale_color_manual(values=c("#999999", "#FF9600"))

 # + scale_y_continuous(breaks = -4:9, label=forest_vep$label)

  + coord_cartesian(xlim=c(-1.1, 0.5))
  + scale_x_continuous(breaks=seq(-1, 1, 0.5))

  #+ scale_color_manual(values=c("#999999", "#00BFC4", "#F8766D", "#00BFC4", "#F8766D"))

  + labs(x = expression(beta~"for P100 latency reduction (ms / year)"), y = "",
       title = "",
       subtitle = "")
  + theme(legend.position = "none", plot.title = element_blank(), plot.subtitle = element_blank())
  )

forest_vep_subfig_noON


pdf(file="OutputForPaper/fig_forest_all.pdf", width=3, height=2.5)
forest_vep_subfig_all
forest_vep_subfig_noON
dev.off()
```

7. MTR Outcomes

NB: CGM = 4 DGM = 5 Brainstem = 7

CGM

```
modelcgm_bex_ca <- lmer(ch_wles_mtr ~ active.switched:les_location.switched04:Age.frac
                              + active.switched:les_location.switched04 #CGM = baseline
                              + active.switched:Age.frac
                              + les_location.switched04:Age.frac
                              + active.switched
                              + les_location.switched04
                              + bl_wholeles_mtr
                              + bin_edss
                              + gender
                              + site
                              + Age.frac
                              + (1 | id),
                              data = bline_ITT)

modelcgm_plac_ca <- update(modelcgm_bex_ca, . ~ . - active.switched - active.switched:Age.frac - active.switched:les_location.switched04
                           - active.switched:les_location.switched04:Age.frac
                           + active + active:Age.frac + active:les_location.switched04
                           + active:les_location.switched04:Age.frac)

#Brain age (unadjusted)
modelcgm_bex_brainage_raw <- update(modelcgm_bex_ca, . ~ . - Age.frac - active.switched:Age.frac - les_location.switched04:Age.frac
                           - active.switched:les_location.switched04:Age.frac
                           + brainage + active.switched:brainage + les_location.switched04:brainage
                           + active.switched:les_location.switched04:brainage)

#Blood age (unadjusted)
modelcgm_bex_kdm_raw <- update(modelcgm_bex_ca, . ~ . - Age.frac - active.switched:Age.frac - les_location.switched04:Age.frac
                           - active.switched:les_location.switched04:Age.frac
                           + kdm + active.switched:kdm + les_location.switched04:kdm
                           + active.switched:les_location.switched04:kdm)

#Brain age ADVANCEMENT (adjusted)
modelcgm_bex_brainage_adj <- update(modelcgm_bex_ca, . ~ . + brainage_advance + active.switched:brainage_advance + les_location.switched
04:brainage_advance
                           + active.switched:les_location.switched04:brainage_advance)

#Blood age ADVANCEMENT (adjusted)
modelcgm_bex_kdm_adj <- update(modelcgm_bex_ca, . ~ . + kdm_advance + active.switched:kdm_advance + les_location.switched04:kdm_advance
                           + active.switched:les_location.switched04:kdm_advance)
```

DGM

```
modeldgm_bex_ca <- lmer(ch_wles_mtr ~ active.switched:les_location.switched05:Age.frac
                              + active.switched:les_location.switched05 #DGM = baseline
                              + active.switched:Age.frac
                              + les_location.switched05:Age.frac
                              + active.switched
                              + les_location.switched05
                              + bl_wholeles_mtr
                              + bin_edss
                              + gender
                              + site
                              + Age.frac
                              + (1 | id),
                              data = bline_ITT)

modeldgm_plac_ca <- update(modeldgm_bex_ca, . ~ . - active.switched - active.switched:Age.frac - active.switched:les_location.switched05
                           - active.switched:les_location.switched05:Age.frac
                           + active + active:Age.frac + active:les_location.switched05
                           + active:les_location.switched05:Age.frac)

#Brain age (unadjusted)
modeldgm_bex_brainage_raw <- update(modeldgm_bex_ca, . ~ . - Age.frac - active.switched:Age.frac - les_location.switched05:Age.frac
                           - active.switched:les_location.switched05:Age.frac
                           + brainage + active.switched:brainage + les_location.switched05:brainage
                           + active.switched:les_location.switched05:brainage)

#Blood age (unadjusted)
modeldgm_bex_kdm_raw <- update(modeldgm_bex_ca, . ~ . - Age.frac - active.switched:Age.frac - les_location.switched05:Age.frac
                           - active.switched:les_location.switched05:Age.frac
                           + kdm + active.switched:kdm + les_location.switched05:kdm
                           + active.switched:les_location.switched05:kdm)

#Brain age ADVANCEMENT (adjusted)
modeldgm_bex_brainage_adj <- update(modeldgm_bex_ca, . ~ . + brainage_advance + active.switched:brainage_advance + les_location.switched
05:brainage_advance
                           + active.switched:les_location.switched05:brainage_advance)

#Blood age ADVANCEMENT (adjusted)
modeldgm_bex_kdm_adj <- update(modeldgm_bex_ca, . ~ . + kdm_advance + active.switched:kdm_advance + les_location.switched05:kdm_advance
                           + active.switched:les_location.switched05:kdm_advance)
```

Brainstem

```
modelbst_bex_ca <- lmer(ch_wles_mtr ~ active.switched:les_location.switched07:Age.frac
                              + active.switched:les_location.switched07 #Brainstem = baseline
                              + active.switched:Age.frac
                              + les_location.switched07:Age.frac
                              + active.switched
                              + les_location.switched07
                              + bl_wholeles_mtr
                              + bin_edss
                              + gender
                              + site
                              + Age.frac
                              + (1 | id),
                              data = bline_ITT)

modelbst_plac_ca <- update(modelbst_bex_ca, . ~ . - active.switched - active.switched:Age.frac - active.switched:les_location.switched07
                           - active.switched:les_location.switched07:Age.frac
                           + active + active:Age.frac + active:les_location.switched07
                           + active:les_location.switched07:Age.frac)

#Brain age (unadjusted)
modelbst_bex_brainage_raw <- update(modelbst_bex_ca, . ~ . - Age.frac - active.switched:Age.frac - les_location.switched07:Age.frac
                           - active.switched:les_location.switched07:Age.frac
                           + brainage + active.switched:brainage + les_location.switched07:brainage
                           + active.switched:les_location.switched07:brainage)

#Blood age (unadjusted)
modelbst_bex_kdm_raw <- update(modelbst_bex_ca, . ~ . - Age.frac - active.switched:Age.frac - les_location.switched07:Age.frac
                           - active.switched:les_location.switched07:Age.frac
                           + kdm + active.switched:kdm + les_location.switched07:kdm
                           + active.switched:les_location.switched07:kdm)

#Brain age ADVANCEMENT (adjusted)
modelbst_bex_brainage_adj <- update(modelbst_bex_ca, . ~ . + brainage_advance + active.switched:brainage_advance + les_location.switched
07:brainage_advance
                           + active.switched:les_location.switched07:brainage_advance)

#Blood age ADVANCEMENT (adjusted)
modelbst_bex_kdm_adj <- update(modelbst_bex_ca, . ~ . + kdm_advance + active.switched:kdm_advance + les_location.switched07:kdm_advance
                           + active.switched:les_location.switched07:kdm_advance)
```

Tabulate MTR outcome data: Supplementary Table 6

```
#CGM
forest_cgm <- data.frame(criteria="", label = "", beta="", CI.low="", CI.high="", p="") %>%

rbind(c("ca", " ",
  summary(modelcgm_bex_ca)$coefficients["Age.frac", "Estimate"],
  (summary(modelcgm_bex_ca)$coefficients["Age.frac", "Estimate"] - (1.96 * summary(modelcgm_bex_ca)$coefficients["Age.frac", "Std. Error
"])),
  (summary(modelcgm_bex_ca)$coefficients["Age.frac", "Estimate"] + (1.96 * summary(modelcgm_bex_ca)$coefficients["Age.frac", "Std. Error
```

```
"])),
  summary(modelcgm_bex_ca)$coefficients["Age.frac", "Pr(>|t|)"])) %>%

  rbind(data.frame(criteria="blank1", label = "", beta=NA, CI.low=NA, CI.high=NA, p=NA)) %>%

  rbind(c("brain_raw", "Unadjusted",
  summary(modelcgm_bex_brainage_raw)$coefficients["brainage", "Estimate"],
  (summary(modelcgm_bex_brainage_raw)$coefficients["brainage", "Estimate"] - (1.96 * summary(modelcgm_bex_brainage_raw)$coefficients["br
ainage", "Std. Error"])),
  (summary(modelcgm_bex_brainage_raw)$coefficients["brainage", "Estimate"] + (1.96 * summary(modelcgm_bex_brainage_raw)$coefficients["br
ainage", "Std. Error"])),
  summary(modelcgm_bex_brainage_raw)$coefficients["brainage", "Pr(>|t|)"])) %>%

  rbind(c("brain_adj", "Adjusted",
  summary(modelcgm_bex_brainage_adj)$coefficients["brainage_advance", "Estimate"],
  (summary(modelcgm_bex_brainage_adj)$coefficients["brainage_advance", "Estimate"] - (1.96 * summary(modelcgm_bex_brainage_adj)$coeffici
ents["brainage_advance", "Std. Error"])),
  (summary(modelcgm_bex_brainage_adj)$coefficients["brainage_advance", "Estimate"] + (1.96 * summary(modelcgm_bex_brainage_adj)$coeffici
ents["brainage_advance", "Std. Error"])),
  summary(modelcgm_bex_brainage_adj)$coefficients["brainage_advance", "Pr(>|t|)"])) %>%

  rbind(data.frame(criteria="blank2", label = "", beta=NA, CI.low=NA, CI.high=NA, p=NA)) %>%

  rbind(c("kdm_raw", "Unadjusted",
  summary(modelcgm_bex_kdm_raw)$coefficients["kdm", "Estimate"],
  (summary(modelcgm_bex_kdm_raw)$coefficients["kdm", "Estimate"] - (1.96 * summary(modelcgm_bex_kdm_raw)$coefficients["kdm", "Std. Error
"])),
  (summary(modelcgm_bex_kdm_raw)$coefficients["kdm", "Estimate"] + (1.96 * summary(modelcgm_bex_kdm_raw)$coefficients["kdm", "Std. Error
"])),
  summary(modelcgm_bex_kdm_raw)$coefficients["kdm", "Pr(>|t|)"])) %>%

  rbind(c("kdm_adj", "Adjusted",
  summary(modelcgm_bex_kdm_adj)$coefficients["kdm_advance", "Estimate"],
  (summary(modelcgm_bex_kdm_adj)$coefficients["kdm_advance", "Estimate"] - (1.96 * summary(modelcgm_bex_kdm_adj)$coefficients["kdm_advan
ce", "Std. Error"])),
  (summary(modelcgm_bex_kdm_adj)$coefficients["kdm_advance", "Estimate"] + (1.96 * summary(modelcgm_bex_kdm_adj)$coefficients["kdm_advan
ce", "Std. Error"])),
  summary(modelcgm_bex_kdm_adj)$coefficients["kdm_advance", "Pr(>|t|)"]))

forest_cgm <- forest_cgm[-1,] %>% mutate_at(c("beta", "CI.low", "CI.high", "p"), as.numeric)
rownames(forest_cgm) <- NULL
forest_cgm$criteria <- factor(forest_cgm$criteria, levels = rev(forest_cgm$criteria))

forest_cgm

###

#DGM
forest_dgm <- data.frame(criteria="", label = "", beta="", CI.low="", CI.high="", p="") %>%

rbind(c("ca", " ",
  summary(modeldgm_bex_ca)$coefficients["Age.frac", "Estimate"],
  (summary(modeldgm_bex_ca)$coefficients["Age.frac", "Estimate"] - (1.96 * summary(modeldgm_bex_ca)$coefficients["Age.frac", "Std. Error
"])),
  (summary(modeldgm_bex_ca)$coefficients["Age.frac", "Estimate"] + (1.96 * summary(modeldgm_bex_ca)$coefficients["Age.frac", "Std. Error
"])),
  summary(modeldgm_bex_ca)$coefficients["Age.frac", "Pr(>|t|)"])) %>%

  rbind(data.frame(criteria="blank1", label = "", beta=NA, CI.low=NA, CI.high=NA, p=NA)) %>%

  rbind(c("brain_raw", "Unadjusted",
  summary(modeldgm_bex_brainage_raw)$coefficients["brainage", "Estimate"],
  (summary(modeldgm_bex_brainage_raw)$coefficients["brainage", "Estimate"] - (1.96 * summary(modeldgm_bex_brainage_raw)$coefficients["br
ainage", "Std. Error"])),
  (summary(modeldgm_bex_brainage_raw)$coefficients["brainage", "Estimate"] + (1.96 * summary(modeldgm_bex_brainage_raw)$coefficients["br
ainage", "Std. Error"])),
  summary(modeldgm_bex_brainage_raw)$coefficients["brainage", "Pr(>|t|)"])) %>%

  rbind(c("brain_adj", "Adjusted",
  summary(modeldgm_bex_brainage_adj)$coefficients["brainage_advance", "Estimate"],
  (summary(modeldgm_bex_brainage_adj)$coefficients["brainage_advance", "Estimate"] - (1.96 * summary(modeldgm_bex_brainage_adj)$coeffici
ents["brainage_advance", "Std. Error"])),
  (summary(modeldgm_bex_brainage_adj)$coefficients["brainage_advance", "Estimate"] + (1.96 * summary(modeldgm_bex_brainage_adj)$coeffici
ents["brainage_advance", "Std. Error"])),
  summary(modeldgm_bex_brainage_adj)$coefficients["brainage_advance", "Pr(>|t|)"])) %>%

  rbind(data.frame(criteria="blank2", label = "", beta=NA, CI.low=NA, CI.high=NA, p=NA)) %>%

  rbind(c("kdm_raw", "Unadjusted",
  summary(modeldgm_bex_kdm_raw)$coefficients["kdm", "Estimate"],
  (summary(modeldgm_bex_kdm_raw)$coefficients["kdm", "Estimate"] - (1.96 * summary(modeldgm_bex_kdm_raw)$coefficients["kdm", "Std. Error
"])),
  (summary(modeldgm_bex_kdm_raw)$coefficients["kdm", "Estimate"] + (1.96 * summary(modeldgm_bex_kdm_raw)$coefficients["kdm", "Std. Error
"])),
  summary(modeldgm_bex_kdm_raw)$coefficients["kdm", "Pr(>|t|)"])) %>%

  rbind(c("kdm_adj", "Adjusted",
  summary(modeldgm_bex_kdm_adj)$coefficients["kdm_advance", "Estimate"],
  (summary(modeldgm_bex_kdm_adj)$coefficients["kdm_advance", "Estimate"] - (1.96 * summary(modeldgm_bex_kdm_adj)$coefficients["kdm_advan
ce", "Std. Error"])),
  (summary(modeldgm_bex_kdm_adj)$coefficients["kdm_advance", "Estimate"] + (1.96 * summary(modeldgm_bex_kdm_adj)$coefficients["kdm_advan
ce", "Std. Error"])),
  summary(modeldgm_bex_kdm_adj)$coefficients["kdm_advance", "Pr(>|t|)"]))

forest_dgm <- forest_dgm[-1,] %>% mutate_at(c("beta", "CI.low", "CI.high", "p"), as.numeric)
rownames(forest_dgm) <- NULL
forest_dgm$criteria <- factor(forest_dgm$criteria, levels = rev(forest_dgm$criteria))

forest_dgm
```

```
###

#Brainstem
forest_bst <- data.frame(criteria="", label = "", beta="", CI.low="", CI.high="", p="") %>%

rbind(c("ca", " ",
  summary(modelbst_bex_ca)$coefficients["Age.frac", "Estimate"],
  (summary(modelbst_bex_ca)$coefficients["Age.frac", "Estimate"] - (1.96 * summary(modelbst_bex_ca)$coefficients["Age.frac", "Std. Error
"])),
  (summary(modelbst_bex_ca)$coefficients["Age.frac", "Estimate"] + (1.96 * summary(modelbst_bex_ca)$coefficients["Age.frac", "Std. Error
"])),
  summary(modelbst_bex_ca)$coefficients["Age.frac", "Pr(>|t|)"])) %>%

  rbind(data.frame(criteria="blank1", label = "", beta=NA, CI.low=NA, CI.high=NA, p=NA)) %>%

  rbind(c("brain_raw", "Unadjusted",
  summary(modelbst_bex_brainage_raw)$coefficients["brainage", "Estimate"],
  (summary(modelbst_bex_brainage_raw)$coefficients["brainage", "Estimate"] - (1.96 * summary(modelbst_bex_brainage_raw)$coefficients["br
ainage", "Std. Error"])),
  (summary(modelbst_bex_brainage_raw)$coefficients["brainage", "Estimate"] + (1.96 * summary(modelbst_bex_brainage_raw)$coefficients["br
ainage", "Std. Error"])),
  summary(modelbst_bex_brainage_raw)$coefficients["brainage", "Pr(>|t|)"])) %>%

  rbind(c("brain_adj", "Adjusted",
  summary(modelbst_bex_brainage_adj)$coefficients["brainage_advance", "Estimate"],
  (summary(modelbst_bex_brainage_adj)$coefficients["brainage_advance", "Estimate"] - (1.96 * summary(modelbst_bex_brainage_adj)$coeffici
ents["brainage_advance", "Std. Error"])),
  (summary(modelbst_bex_brainage_adj)$coefficients["brainage_advance", "Estimate"] + (1.96 * summary(modelbst_bex_brainage_adj)$coeffici
ents["brainage_advance", "Std. Error"])),
  summary(modelbst_bex_brainage_adj)$coefficients["brainage_advance", "Pr(>|t|)"])) %>%

  rbind(data.frame(criteria="blank2", label = "", beta=NA, CI.low=NA, CI.high=NA, p=NA)) %>%

  rbind(c("kdm_raw", "Unadjusted",
  summary(modelbst_bex_kdm_raw)$coefficients["kdm", "Estimate"],
  (summary(modelbst_bex_kdm_raw)$coefficients["kdm", "Estimate"] - (1.96 * summary(modelbst_bex_kdm_raw)$coefficients["kdm", "Std. Error
"])),
  (summary(modelbst_bex_kdm_raw)$coefficients["kdm", "Estimate"] + (1.96 * summary(modelbst_bex_kdm_raw)$coefficients["kdm", "Std. Error
"])),
  summary(modelbst_bex_kdm_raw)$coefficients["kdm", "Pr(>|t|)"])) %>%

  rbind(c("kdm_adj", "Adjusted",
  summary(modelbst_bex_kdm_adj)$coefficients["kdm_advance", "Estimate"],
  (summary(modelbst_bex_kdm_adj)$coefficients["kdm_advance", "Estimate"] - (1.96 * summary(modelbst_bex_kdm_adj)$coefficients["kdm_advan
ce", "Std. Error"])),
  (summary(modelbst_bex_kdm_adj)$coefficients["kdm_advance", "Estimate"] + (1.96 * summary(modelbst_bex_kdm_adj)$coefficients["kdm_advan
ce", "Std. Error"])),
  summary(modelbst_bex_kdm_adj)$coefficients["kdm_advance", "Pr(>|t|)"]))

forest_bst <- forest_bst[-1,] %>% mutate_at(c("beta", "CI.low", "CI.high", "p"), as.numeric)
rownames(forest_bst) <- NULL
forest_bst$criteria <- factor(forest_bst$criteria, levels = rev(forest_bst$criteria))

forest_bst

###
```

MTR plot: FIG 4

```
forest_cgm2 <- forest_cgm
forest_cgm2$shape <- forest_vep_all$shape
forest_cgm2$label <- c(" ", "", "Unadj.", "Adj.", "", "Unadj.", "Adj.")

forest_dgm2 <- forest_dgm
forest_dgm2$shape <- forest_vep_all$shape
forest_dgm2$label <- c(" ", "", "Unadj.", "Adj.", "", "Unadj.", "Adj.")

forest_bst2 <- forest_bst
forest_bst2$shape <- forest_vep_all$shape
forest_bst2$label <- c(" ", "", "Unadj.", "Adj.", "", "Unadj.", "Adj.")

forest_cgm_subfig2 <- (ggplot(data = forest_cgm2, aes(y=criteria, x=beta, xmin=CI.low, xmax=CI.high, colour=p<0.05))
  + theme_classic()
  + geom_vline(xintercept=0, colour="DarkGray")
  + geom_hline(yintercept="blank1", colour="#999999", linetype = "dashed")
  + geom_hline(yintercept="blank2", colour="#999999", linetype = "dashed")

  + geom_point(aes(shape=forest_cgm2$shape))
  + scale_shape_manual(values=c(0,16,17, 0))
  + geom_errorbar(height=.1)

  + scale_y_discrete(label=rev(forest_cgm2$label))
  + scale_color_manual(values=c("#999999", "#FF9600"))

  + coord_cartesian(xlim=c(-0.2, 0.2))
  + scale_x_continuous(breaks=seq(-0.2, 0.2, 0.1))

  + labs(x = expression(beta~"for change in MTR (pu / year)"), y = "",
      title = "",
      subtitle = "")
  + theme(legend.position = "none", plot.title = element_blank(), plot.subtitle = element_blank())
  )

forest_cgm_subfig2
```

```r
forest_dgm_subfig2 <- (ggplot(data = forest_dgm2, aes(y=criteria, x=beta, xmin=CI.low, xmax=CI.high, colour=p<0.05))
  + theme_classic()
  + geom_vline(xintercept=0, colour="DarkGray")
  + geom_hline(yintercept="blank1", colour="#999999", linetype = "dashed")
  + geom_hline(yintercept="blank2", colour="#999999", linetype = "dashed")

  + geom_point(aes(shape=forest_dgm2$shape))
  + scale_shape_manual(values=c(0,16,17, 0))
  + geom_errorbarh(height=.1)

  + scale_y_discrete(label=rev(forest_dgm2$label))
  + scale_color_manual(values=c("#999999", "#FF9600"))

  + coord_cartesian(xlim=c(-0.6, 0.6))
  + scale_x_continuous(breaks=seq(-0.5, 0.5, 0.5))

  + labs(x = expression(beta~"for change in MTR (pu / year)"), y = "",
        title = "",
        subtitle = "")
  + theme(legend.position = "none", plot.title = element_blank(), plot.subtitle = element_blank())
  )

forest_dgm_subfig2

forest_bst_subfig2 <- (ggplot(data = forest_bst2, aes(y=criteria, x=beta, xmin=CI.low, xmax=CI.high, colour=p<0.05))
  + theme_classic()
  + geom_vline(xintercept=0, colour="DarkGray")
  + geom_hline(yintercept="blank1", colour="#999999", linetype = "dashed")
  + geom_hline(yintercept="blank2", colour="#999999", linetype = "dashed")

  + geom_point(aes(shape=forest_bst2$shape))
  + scale_shape_manual(values=c(0,16,17, 0))
  + geom_errorbarh(height=.1)

  + scale_y_discrete(label=rev(forest_bst2$label))
  + scale_color_manual(values=c("#999999", "#FF9600"))

  + coord_cartesian(xlim=c(-0.2, 0.2))
  + scale_x_continuous(breaks=seq(-0.2, 0.2, 0.1))

  + labs(x = expression(beta~"for change in MTR (pu / year)"), y = "",
        title = "",
        subtitle = "")
  + theme(legend.position = "none", plot.title = element_blank(), plot.subtitle = element_blank())
  )

forest_bst_subfig2

pdf(file="OutputForPaper/fig_mtr_forest_all.pdf", width=2.5, height=2.5)
forest_cgm_subfig2
forest_dgm_subfig2
forest_bst_subfig2
dev.off()
```

8. Repeat with lymphocytes excluded

VEP with lymphocytes excluded: Supplementary Table 7

```r
#KDM age (unadjusted)
modelvep_bex_kdmnl_raw <- update(modelvep_bex_ca, . ~ . - Age.frac - active.switched:Age.frac - bin_bline_P100.switched01:Age.frac
                            - active.switched:bin_bline_P100.switched01:Age.frac
                            + kdm_no_lymph + active.switched:kdm_no_lymph + bin_bline_P100.switched01:kdm_no_lymph
                            + active.switched:bin_bline_P100.switched01:kdm_no_lymph)

diff <- summary(modelvep_bex_kdmnl_raw)$coefficients["kdm_no_lymph","Estimate"]
SE <- summary(modelvep_bex_kdmnl_raw)$coefficients["kdm_no_lymph","Std. Error"]
p <- summary(modelvep_bex_kdmnl_raw)$coefficients["kdm_no_lymph","Pr(>|t|)"]
print(sprintf("Unadjusted blood-based BA age effect (w/o lymphocytes): %.02f ms/year [CI: %.02f to %.02f], p = %.03f", diff, (diff-1.96*
SE), (diff+1.96*SE), p))


modelvep_bex_kdmnl_raw_exc <- update(modelvep_bex_kdmnl_raw, ch_veplat100_no5yON ~ .)

diff <- summary(modelvep_bex_kdmnl_raw_exc)$coefficients["kdm_no_lymph","Estimate"]
SE <- summary(modelvep_bex_kdmnl_raw_exc)$coefficients["kdm_no_lymph","Std. Error"]
p <- summary(modelvep_bex_kdmnl_raw_exc)$coefficients["kdm_no_lymph","Pr(>|t|)"]
print(sprintf("Unadjusted blood-based BA age effect (w/o lymphocytes; ON excluded): %.02f ms/year [CI: %.02f to %.02f], p = %.03f", diff
, (diff-1.96*SE), (diff+1.96*SE), p))


#KDM age ADVANCEMENT (adjusted)
modelvep_bex_kdmnl_adj <- update(modelvep_bex_ca, . ~ . + kdm_no_lymph_advance + active.switched:kdm_no_lymph_advance + bin_bline_P100.s
witched01:kdm_no_lymph_advance
                            + active.switched:bin_bline_P100.switched01:kdm_no_lymph_advance)
diff <- summary(modelvep_bex_kdmnl_adj)$coefficients["kdm_no_lymph_advance","Estimate"]
SE <- summary(modelvep_bex_kdmnl_adj)$coefficients["kdm_no_lymph_advance","Std. Error"]
p <- summary(modelvep_bex_kdmnl_adj)$coefficients["kdm_no_lymph_advance","Pr(>|t|)"]
print(sprintf("Adjusted blood-based BA age advancement effect (w/o lymphocytes): %.02f ms/year [CI: %.02f to %.02f], p = %.03f", diff, (
diff-1.96*SE), (diff+1.96*SE), p))

modelvep_bex_kdmnl_adj_exc <- update(modelvep_bex_kdmnl_adj, ch_veplat100_no5yON ~ .)
diff <- summary(modelvep_bex_kdmnl_adj_exc)$coefficients["kdm_no_lymph_advance","Estimate"]
SE <- summary(modelvep_bex_kdmnl_adj_exc)$coefficients["kdm_no_lymph_advance","Std. Error"]
```

```
p <- summary(modelvep_bex_kdmnl_adj_exc)$coefficients["kdm_no_lymph_advance","Pr(>|t|)"]
print(sprintf("Adjusted blood-based BA age advancement effect (w/o lymphocytes; ON excluded): %.02f ms/year [CI: %.02f to %.02f], p = %.
03f", diff, (diff-1.96*SE), (diff+1.96*SE), p))

#[N.B. all sign-reversed as per previous models, as a negative effect on P100 is a positive outcome]
```

MTR with lymphocytes excluded: Supplementary Table 8

```
#CGM
print("CGM")

#KDM age (unadjusted)
modelcgm_bex_kdmnl_raw <- update(modelcgm_bex_ca, . ~ . - Age.frac - active.switched:Age.frac - les_location.switched04:Age.frac
                           - active.switched:les_location.switched04:Age.frac
                           + kdm_no_lymph + active.switched:kdm_no_lymph + les_location.switched04:kdm_no_lymph
                           + active.switched:les_location.switched04:kdm_no_lymph)

diff <- summary(modelcgm_bex_kdmnl_raw)$coefficients["kdm_no_lymph","Estimate"]
SE <- summary(modelcgm_bex_kdmnl_raw)$coefficients["kdm_no_lymph","Std. Error"]
p <- summary(modelcgm_bex_kdmnl_raw)$coefficients["kdm_no_lymph","Pr(>|t|)"]
print(sprintf("Unadjusted blood-based BA age effect (w/o lymphocytes): %.02f pu/year [CI: %.02f to %.02f], p = %.03f", diff, (diff-1.96*
SE), (diff+1.96*SE), p))

#KDM age ADVANCEMENT (adjusted)
modelcgm_bex_kdmnl_adj <- update(modelcgm_bex_ca, . ~ . + kdm_no_lymph_advance + active.switched:kdm_no_lymph_advance + les_location.swi
tched04:kdm_no_lymph_advance
                           + active.switched:les_location.switched04:kdm_no_lymph_advance)
diff <- summary(modelcgm_bex_kdmnl_adj)$coefficients["kdm_no_lymph_advance","Estimate"]
SE <- summary(modelcgm_bex_kdmnl_adj)$coefficients["kdm_no_lymph_advance","Std. Error"]
p <- summary(modelcgm_bex_kdmnl_adj)$coefficients["kdm_no_lymph_advance","Pr(>|t|)"]
print(sprintf("Adjusted blood-based BA age advancement effect (w/o lymphocytes): %.02f ms/year [CI: %.02f to %.02f], p = %.03f", diff, (
diff-1.96*SE), (diff+1.96*SE), p))


#DGM
print("")
print("DGM")

#KDM age (unadjusted)
modeldgm_bex_kdmnl_raw <- update(modeldgm_bex_ca, . ~ . - Age.frac - active.switched:Age.frac - les_location.switched05:Age.frac
                           - active.switched:les_location.switched05:Age.frac
                           + kdm_no_lymph + active.switched:kdm_no_lymph + les_location.switched05:kdm_no_lymph
                           + active.switched:les_location.switched05:kdm_no_lymph)

diff <- summary(modeldgm_bex_kdmnl_raw)$coefficients["kdm_no_lymph","Estimate"]
SE <- summary(modeldgm_bex_kdmnl_raw)$coefficients["kdm_no_lymph","Std. Error"]
p <- summary(modeldgm_bex_kdmnl_raw)$coefficients["kdm_no_lymph","Pr(>|t|)"]
print(sprintf("Unadjusted blood-based BA age effect (w/o lymphocytes): %.02f pu/year [CI: %.02f to %.02f], p = %.03f", diff, (diff-1.96*
SE), (diff+1.96*SE), p))

#KDM age ADVANCEMENT (adjusted)
modeldgm_bex_kdmnl_adj <- update(modeldgm_bex_ca, . ~ . + kdm_no_lymph_advance + active.switched:kdm_no_lymph_advance + les_location.swi
tched05:kdm_no_lymph_advance
                           + active.switched:les_location.switched05:kdm_no_lymph_advance)
diff <- summary(modeldgm_bex_kdmnl_adj)$coefficients["kdm_no_lymph_advance","Estimate"]
SE <- summary(modeldgm_bex_kdmnl_adj)$coefficients["kdm_no_lymph_advance","Std. Error"]
p <- summary(modeldgm_bex_kdmnl_adj)$coefficients["kdm_no_lymph_advance","Pr(>|t|)"]
print(sprintf("Adjusted blood-based BA age advancement effect (w/o lymphocytes): %.02f ms/year [CI: %.02f to %.02f], p = %.03f", diff, (
diff-1.96*SE), (diff+1.96*SE), p))


#Brainstem
print("")
print("Brainstem")

#KDM age (unadjusted)
modelbst_bex_kdmnl_raw <- update(modelbst_bex_ca, . ~ . - Age.frac - active.switched:Age.frac - les_location.switched07:Age.frac
                           - active.switched:les_location.switched07:Age.frac
                           + kdm_no_lymph + active.switched:kdm_no_lymph + les_location.switched07:kdm_no_lymph
                           + active.switched:les_location.switched07:kdm_no_lymph)

diff <- summary(modelbst_bex_kdmnl_raw)$coefficients["kdm_no_lymph","Estimate"]
SE <- summary(modelbst_bex_kdmnl_raw)$coefficients["kdm_no_lymph","Std. Error"]
p <- summary(modelbst_bex_kdmnl_raw)$coefficients["kdm_no_lymph","Pr(>|t|)"]
print(sprintf("Unadjusted blood-based BA age effect (w/o lymphocytes): %.02f pu/year [CI: %.02f to %.02f], p = %.03f", diff, (diff-1.96*
SE), (diff+1.96*SE), p))

#KDM age ADVANCEMENT (adjusted)
modelbst_bex_kdmnl_adj <- update(modelbst_bex_ca, . ~ . + kdm_no_lymph_advance + active.switched:kdm_no_lymph_advance + les_location.swi
tched07:kdm_no_lymph_advance
                           + active.switched:les_location.switched07:kdm_no_lymph_advance)
diff <- summary(modelbst_bex_kdmnl_adj)$coefficients["kdm_no_lymph_advance","Estimate"]
SE <- summary(modelbst_bex_kdmnl_adj)$coefficients["kdm_no_lymph_advance","Std. Error"]
p <- summary(modelbst_bex_kdmnl_adj)$coefficients["kdm_no_lymph_advance","Pr(>|t|)"]
print(sprintf("Adjusted blood-based BA age advancement effect (w/o lymphocytes): %.02f ms/year [CI: %.02f to %.02f], p = %.03f", diff, (
diff-1.96*SE), (diff+1.96*SE), p))
```

9. Diffuse MTR changes: FIG S1

```
# MTR changes
brainage_all$CGMmtr.change <- brainage_all$CGMmtr.6 - brainage_all$CGMmtr.0
brainage_all$brainstemmtr.change <- brainage_all$brainstemmtr.6 - brainage_all$brainstemmtr.0
```

```
#CGM
cgm_change_mtr_summary_data_exc <- brainage_all[!brainage_all$patient_id %in% new_lesion_ids,c("active", "CGMmtr.change")] %>%
                 group_by(active) %>%
                 dplyr::summarise(mean = mean(CGMmtr.change)) %>%
  merge(brainage_all[!brainage_all$patient_id %in% new_lesion_ids,c("active", "CGMmtr.change")] %>%
                 group_by(active) %>%
                 dplyr::summarise(sem = (sd(CGMmtr.change)/sqrt(length(CGMmtr.change)))),
        by = "active") %>%
  merge(brainage_all[!brainage_all$patient_id %in% new_lesion_ids,c("active", "CGMmtr.change")] %>%
                 group_by(active) %>%
                 dplyr::summarise(sd = (sd(CGMmtr.change))),
        by = "active")

cgm_change_mtr_summary_data_exc$CI_low <- cgm_change_mtr_summary_data_exc$mean - 1.96 * cgm_change_mtr_summary_data_exc$sem
cgm_change_mtr_summary_data_exc$CI_high <- cgm_change_mtr_summary_data_exc$mean + 1.96 * cgm_change_mtr_summary_data_exc$sem


fig_cgm_change_mtr_short <- (ggplot()
  + theme_classic()
  + geom_hline(yintercept=0, colour="DarkGray")

  + geom_point(data=brainage_all[!brainage_all$patient_id %in% new_lesion_ids,], aes(x = active, y = CGMmtr.change, group = patient_id,
colour = active), alpha =0.6, position = position_jitter(width = 0.1, seed = 0))

  + geom_errorbar(data=cgm_change_mtr_summary_data_exc, aes(x = active, ymin=CI_low, ymax=CI_high), colour="black", width=.08)
  + geom_point(data=cgm_change_mtr_summary_data_exc, aes(x = active, y = mean), colour="black", alpha =1, shape=18, size = 2, fill = "wh
ite")

  + labs(title = "", subtitle = "", x = "", y = "Change in non-lesion\nCGM MTR (pu)")
  + theme(legend.position = "none", plot.title = element_text(hjust = 0.5), plot.subtitle = element_text(hjust = 0.5))
  + coord_cartesian(ylim = c(-2, 2),)
  + scale_y_continuous(breaks=seq(-2,2,1))
)

# Brainstem
brainstem_change_mtr_summary_data_exc <- brainage_all[!brainage_all$patient_id %in% new_lesion_ids,c("active", "brainstemmtr.change")] %
>%
                 group_by(active) %>%
                 dplyr::summarise(mean = mean(brainstemmtr.change)) %>%
  merge(brainage_all[!brainage_all$patient_id %in% new_lesion_ids,c("active", "brainstemmtr.change")] %>%
                 group_by(active) %>%
                 dplyr::summarise(sem = (sd(brainstemmtr.change)/sqrt(length(brainstemmtr.change)))),
        by = "active") %>%
  merge(brainage_all[!brainage_all$patient_id %in% new_lesion_ids,c("active", "brainstemmtr.change")] %>%
                 group_by(active) %>%
                 dplyr::summarise(sd = (sd(brainstemmtr.change))),
        by = "active")

brainstem_change_mtr_summary_data_exc$CI_low <- brainstem_change_mtr_summary_data_exc$mean - 1.96 * brainstem_change_mtr_summary_data_ex
c$sem
brainstem_change_mtr_summary_data_exc$CI_high <- brainstem_change_mtr_summary_data_exc$mean + 1.96 * brainstem_change_mtr_summary_data_e
xc$sem


fig_brainstem_change_mtr_short <- (ggplot()
  + theme_classic()
  + geom_hline(yintercept=0, colour="DarkGray")

  + geom_point(data=brainage_all[!brainage_all$patient_id %in% new_lesion_ids,], aes(x = active, y = brainstemmtr.change, group = patien
t_id, colour = active), alpha =0.6, position = position_jitter(width = 0.1, seed = 0))

  + geom_errorbar(data=brainstem_change_mtr_summary_data_exc, aes(x = active, ymin=CI_low, ymax=CI_high), colour="black", width=.08)
  + geom_point(data=brainstem_change_mtr_summary_data_exc, aes(x = active, y = mean), colour="black", alpha =1, shape=18, size = 2, fill
= "white")

  + labs(title = "", subtitle = "", x = "", y = "Change in non-lesion\nbrainstem MTR (pu)")
  + theme(legend.position = "none", plot.title = element_text(hjust = 0.5), plot.subtitle = element_text(hjust = 0.5))
  + coord_cartesian(ylim = c(-4, 4),)
)

pdf(file="OutputForPaper/fig_change_nlmtr_short.pdf", width=3, height=3)
fig_cgm_change_mtr_short
fig_brainstem_change_mtr_short
dev.off()

fig_cgm_change_mtr_short


fig_brainstem_change_mtr_short
cgm_change_mtr_summary_data_exc
brainstem_change_mtr_summary_data_exc


#Linear model for cgm_mtr change bex vs placebo (adjusting for baseline value)
model_cgm_mtr_change <- lm(CGMmtr.6 ~ active + CGMmtr.0,
     data = brainage_all)

diff <- summary(model_cgm_mtr_change)$coefficients["activeBexarotene","Estimate"]
SE <- summary(model_cgm_mtr_change)$coefficients["activeBexarotene","Std. Error"]
p <- summary(model_cgm_mtr_change)$coefficients["activeBexarotene","Pr(>|t|)"]
print(sprintf("Effect of bexarotene on non-lesion CGM MTR: %.02f pu [CI: %.02f to %.02f], p = %.03f", diff, (diff-1.96*SE), (diff+1.96*S
E), p))


model_cgm_mtr_change_exc <- update(model_cgm_mtr_change, .~., data = brainage_all[!brainage_all$patient_id %in% new_lesion_ids,])

diff <- summary(model_cgm_mtr_change_exc)$coefficients["activeBexarotene","Estimate"]
SE <- summary(model_cgm_mtr_change_exc)$coefficients["activeBexarotene","Std. Error"]
p <- summary(model_cgm_mtr_change_exc)$coefficients["activeBexarotene","Pr(>|t|)"]
print(sprintf("Affect of bexarotene on non-lesion CGM MTR (brains with new lesions excluded): %.02f pu [CI: %.02f to %.02f], p = %.03f",
```

```
diff, (diff-1.96*SE), (diff+1.96*SE), p))

#Linear model for brainstem_mtr change bex vs placebo (adjusting for baseline value)
model_bst_mtr_change <- lm(brainstemmtr.6 ~ active + brainstemmtr.0,
     data = brainage_all)

diff <- summary(model_bst_mtr_change)$coefficients["activeBexarotene","Estimate"]
SE <- summary(model_bst_mtr_change)$coefficients["activeBexarotene","Std. Error"]
p <- summary(model_bst_mtr_change)$coefficients["activeBexarotene","Pr(>|t|)"]
print(sprintf("Effect of bexarotene on non-lesion brainstem MTR: %.02f pu [CI: %.02f to %.02f], p = %.03f", diff, (diff-1.96*SE), (diff+
1.96*SE), p))


model_bst_mtr_change_exc <- update(model_bst_mtr_change, .~., data = brainage_all[!brainage_all$patient_id %in% new_lesion_ids,])

diff <- summary(model_bst_mtr_change_exc)$coefficients["activeBexarotene","Estimate"]
SE <- summary(model_bst_mtr_change_exc)$coefficients["activeBexarotene","Std. Error"]
p <- summary(model_bst_mtr_change_exc)$coefficients["activeBexarotene","Pr(>|t|)"]
print(sprintf("Affect of bexarotene on non-lesion brainstem MTR (brains with new lesions excluded): %.02f pu [CI: %.02f to %.02f], p = %
.03f", diff, (diff-1.96*SE), (diff+1.96*SE), p))
```

10. Volume changes: FIG S2

```
# Volume changes (combine GM volumes)
brainage_all$tGMvol.0 <- brainage_all$CGMvol.0 + brainage_all$DGMvol.0
brainage_all$tGMvol.6 <- brainage_all$CGMvol.6 + brainage_all$DGMvol.6

brainage_all$WMvol.change <- brainage_all$WMvol.6 - brainage_all$WMvol.0
brainage_all$tGMvol.change <- brainage_all$tGMvol.6 - brainage_all$tGMvol.0

#tGM (total GM)
tgm_change_vol_summary_data_exc <- brainage_all[!brainage_all$patient_id %in% new_lesion_ids,c("active", "tGMvol.change")] %>%
                 group_by(active) %>%
                 dplyr::summarise(mean = mean(tGMvol.change)) %>%
  merge(brainage_all[!brainage_all$patient_id %in% new_lesion_ids,c("active", "tGMvol.change")] %>%
                 group_by(active) %>%
                 dplyr::summarise(sem = (sd(tGMvol.change)/sqrt(length(tGMvol.change)))),
        by = "active") %>%
  merge(brainage_all[!brainage_all$patient_id %in% new_lesion_ids,c("active", "tGMvol.change")] %>%
                 group_by(active) %>%
                 dplyr::summarise(sd = (sd(tGMvol.change))),
        by = "active")

tgm_change_vol_summary_data_exc$CI_low <- tgm_change_vol_summary_data_exc$mean - 1.96 * tgm_change_vol_summary_data_exc$sem
tgm_change_vol_summary_data_exc$CI_high <- tgm_change_vol_summary_data_exc$mean + 1.96 * tgm_change_vol_summary_data_exc$sem


fig_tgm_change_vol_short <- (ggplot()
  + theme_classic()
  + geom_hline(yintercept=0, colour="DarkGray")

  + geom_point(data=brainage_all[!brainage_all$patient_id %in% new_lesion_ids,], aes(x = active, y = tGMvol.change, group = patient_id,
colour = active), alpha =0.6, position = position_jitter(width = 0.1, seed = 0))

  + geom_errorbar(data=tgm_change_vol_summary_data_exc, aes(x = active, ymin=CI_low, ymax=CI_high), colour="black", width=.08)
  + geom_point(data=tgm_change_vol_summary_data_exc, aes(x = active, y = mean), colour="black", alpha =1, shape=18, size = 2, fill = "wh
ite")

  + labs(title = "", subtitle = "", x = "", y = "Change in global grey\nmatter volume (mm3)")
  + theme(legend.position = "none", plot.title = element_text(hjust = 0.5), plot.subtitle = element_text(hjust = 0.5))
  + scale_y_continuous(breaks=seq(-20000,10000,5000))
  )

#WM
wm_change_vol_summary_data_exc <- brainage_all[!brainage_all$patient_id %in% new_lesion_ids,c("active", "WMvol.change")] %>%
                 group_by(active) %>%
                 dplyr::summarise(mean = mean(WMvol.change)) %>%
  merge(brainage_all[!brainage_all$patient_id %in% new_lesion_ids,c("active", "WMvol.change")] %>%
                 group_by(active) %>%
                 dplyr::summarise(sem = (sd(WMvol.change)/sqrt(length(WMvol.change)))),
        by = "active") %>%
  merge(brainage_all[!brainage_all$patient_id %in% new_lesion_ids,c("active", "WMvol.change")] %>%
                 group_by(active) %>%
                 dplyr::summarise(sd = (sd(WMvol.change))),
        by = "active")

wm_change_vol_summary_data_exc$CI_low <- wm_change_vol_summary_data_exc$mean - 1.96 * wm_change_vol_summary_data_exc$sem
wm_change_vol_summary_data_exc$CI_high <- wm_change_vol_summary_data_exc$mean + 1.96 * wm_change_vol_summary_data_exc$sem


fig_wm_change_vol_short <- (ggplot()
  + theme_classic()
  + geom_hline(yintercept=0, colour="DarkGray")

  + geom_point(data=brainage_all[!brainage_all$patient_id %in% new_lesion_ids,], aes(x = active, y = WMvol.change, group = patient_id, c
olour = active), alpha =0.6, position = position_jitter(width = 0.1, seed = 0))

  + geom_errorbar(data=wm_change_vol_summary_data_exc, aes(x = active, ymin=CI_low, ymax=CI_high), colour="black", width=.08)
  + geom_point(data=wm_change_vol_summary_data_exc, aes(x = active, y = mean), colour="black", alpha =1, shape=18, size = 2, fill = "whi
te")

  + labs(title = "", subtitle = "", x = "", y = "Change in global white\nmatter volume (mm3)")
  + theme(legend.position = "none", plot.title = element_text(hjust = 0.5), plot.subtitle = element_text(hjust = 0.5))
  + scale_y_continuous(breaks=seq(-10000,15000,5000))
  )
```

```
pdf(file="OutputForPaper/fig_change_vol_short.pdf", width=3, height=3)
fig_tgm_change_vol_short
fig_wm_change_vol_short
dev.off()

fig_tgm_change_vol_short
fig_wm_change_vol_short


tgm_change_vol_summary_data_exc
wm_change_vol_summary_data_exc

#Linear model for tgm_vol change bex vs placebo (adjusting for baseline value and for TIV)
model_tgm_vol_change <- lm(tGMvol.6 ~ active + tGMvol.0 + TIV.0,
     data = brainage_all)

diff <- summary(model_tgm_vol_change)$coefficients["activeBexarotene","Estimate"]
SE <- summary(model_tgm_vol_change)$coefficients["activeBexarotene","Std. Error"]
p <- summary(model_tgm_vol_change)$coefficients["activeBexarotene","Pr(>|t|)"]
print(sprintf("Effect of bexarotene on non-lesion tGM volume: %.02f mm3 [CI: %.02f to %.02f], p = %.03f", diff, (diff-1.96*SE), (diff+1.
96*SE), p))


model_tgm_vol_change_exc <- update(model_tgm_vol_change, .~., data = brainage_all[!brainage_all$patient_id %in% new_lesion_ids,])

diff <- summary(model_tgm_vol_change_exc)$coefficients["activeBexarotene","Estimate"]
SE <- summary(model_tgm_vol_change_exc)$coefficients["activeBexarotene","Std. Error"]
p <- summary(model_tgm_vol_change_exc)$coefficients["activeBexarotene","Pr(>|t|)"]
print(sprintf("Affect of bexarotene on non-lesion tGM volume (brains with new lesions excluded): %.02f mm3 [CI: %.02f to %.02f], p = %.0
3f", diff, (diff-1.96*SE), (diff+1.96*SE), p))


#Linear model for wm_vol change bex vs placebo (adjusting for baseline value and for TIV)
model_wm_vol_change <- lm(WMvol.6 ~ active + WMvol.0 + TIV.0,
     data = brainage_all)

diff <- summary(model_wm_vol_change)$coefficients["activeBexarotene","Estimate"]
SE <- summary(model_wm_vol_change)$coefficients["activeBexarotene","Std. Error"]
p <- summary(model_wm_vol_change)$coefficients["activeBexarotene","Pr(>|t|)"]
print(sprintf("Effect of bexarotene on non-lesion WM volume: %.02f mm3 [CI: %.02f to %.02f], p = %.03f", diff, (diff-1.96*SE), (diff+1.9
6*SE), p))


model_wm_vol_change_exc <- update(model_wm_vol_change, .~., data = brainage_all[!brainage_all$patient_id %in% new_lesion_ids,])

diff <- summary(model_wm_vol_change_exc)$coefficients["activeBexarotene","Estimate"]
SE <- summary(model_wm_vol_change_exc)$coefficients["activeBexarotene","Std. Error"]
p <- summary(model_wm_vol_change_exc)$coefficients["activeBexarotene","Pr(>|t|)"]
print(sprintf("Affect of bexarotene on non-lesion WM volume (brains with new lesions excluded): %.02f mm3 [CI: %.02f to %.02f], p = %.03
f", diff, (diff-1.96*SE), (diff+1.96*SE), p))
```
